# MMP9 integrates multiple immunoregulatory pathways that discriminate high suppressive activity of human mesenchymal stem cells

**DOI:** 10.1038/s41598-017-00923-0

**Published:** 2017-04-13

**Authors:** Carolina Lavini-Ramos, Hernandez Moura Silva, Alessandra Soares-Schanoski, Sandra Maria Monteiro, Ludmila Rodrigues Pinto Ferreira, Ana Paula Pacanaro, Samirah Gomes, Janaína Batista, Kellen Faé, Jorge Kalil, Verônica Coelho

**Affiliations:** 1 Laboratory of Immunology, Heart Institute (InCor), School of Medicine, University of São Paulo, São Paulo, Brazil; 2 Institute for Investigation in Immunology, iii – INCT (National Institute of Science and Technology), São Paulo, Brazil; 3 Center for Cellular and Molecular Therapy Studies (NETCEM), University of São Paulo, São Paulo, Brazil; 4 Universidade Santo Amaro (UNISA), São Paulo, Brazil; 5 Laboratory of Cellular, Genetic, and Molecular Nephrology, Renal Division, University of São Paulo, São Paulo, SP Brazil; 6 Bacterial Vaccines Discovery & Early Development, Crucell Holland B.V., part of the Janssen pharmaceutical companies of Johnson and Johnson, Leiden, The Netherlands; 7 Molecular Pathogenesis Program, Kimmel Center for Biology and Medicine at the Skirball Institute, New York University School of Medicine, New York, NY 10016 USA; 8 Biotechnology Center, Butantan Institute, São Paulo, Brazil

## Abstract

The mechanisms underlying mesenchymal stem cells’ (MSC) suppressive potency are largely unknown. We here show that highly suppressive human adipose tissue-derived MSC (AdMSC) display and induce a differential immunologic profile, upon ongoing AdMSC suppressive activity, promoting: (i) early correlated inhibition of IFN-γ and TNF-α production, along IL-10 increase, (ii) CD73^+^Foxp3^+^Treg subset expansion, and (iii) specific correlations between gene expression increases, such as: MMP9 correlated with CCL22, TNF, FASL, RUNX3, and SEMAD4 in AdMSC and, in T cells, MMP9 upregulation correlated with CCR4, IL4 and TBX21, among others, whereas MMP2 correlated with BCL2 and LRRC31. MMP9 emerged as an integrating molecule for both AdMSC and T cells in molecular networks built with our gene expression data, and we confirmed upregulation of MMP9 and MMP2 at the protein level, in AdMSC and T cells, respectively. MMP2/9 inhibition significantly decreased AdMSC suppressive effect, confirming their important role in suppressive acitivity. We conclude that MMP9 and 2 are robust new players involved in human MSC immunoregulatory mechanisms, and the higher suppressive activity correlates to their capacity to trigger a coordinated action of multiple specific molecules, mobilizing various immunoregulatory mechanisms.

## Introduction

Mesenchymal stem cells (MSC) are multipotent self-renewing stromal cells found in essentially all tissues of the body^1–3^. Due to their multipotenciality and immunoregulatory capacity^4^ MSC have been used experimentally to generate various tissues^5, 6^, for myocardial repair^7^, treating experimental autoimmune encephalomyelitis (EAE)^8^, and graft versus host disease (GVHD)^9^. In clinical trials^10, 11^, MSC have been used to regenerate bone/cartilage^6^, cardiac tissues^7^, to treat diabetes, leukemia, cancers^10^, neurological diseases^10, 12^, GVHD^13–15^, in renal transplantation and for cardiac valve tissue engineering^16^.

MSC affect innate and adaptive immune responses, inhibiting the differentiation of dendritic cells^17^ and B-cells^18^, suppressing proliferation of NK^19^, B^18^ and T cells^20^, and NK cytotoxic activity^21^. Several molecules have been implicated in the immunoregulation mediated by MSC: prostaglandin E2 (PGE2)^4, 20^, indolamine-2,3-dioxygenase (IDO)^20^, leukemia inhibitory factor (LIF)^22^, HLA-G^21, 23^ transforming growth factor-beta (TGF-ß), interleukin 10 (IL-10)^22, 24^ and programmed death 1 ligand (PD-L1)^25^, and inflammatory cytokine-induced intercellular adhesion molecule-1 (ICAM-1) and vascular cell adhesion molecule-1 (VCAM-1)^26^. MSC induce the generation^16, 21^, recruitment and maintenance of regulatory T cells (Treg)^27^, inhibit the differentiation of Th17 cells^28^, downregulate Th1 response^16^ and induce Th2 response^16^. It is currently believed that the inflammatory *milieu*, constituted by IFN-γ^29^, TNF-α, IL-1α or IL-1β^30^, is important to enhance MSC suppressive function.

Despite the well-documented MSC immunoregulatory properties, it is still unclear what triggers their different mechanisms and what determines the heterogeneity in the magnitude of their suppressive capacity. These critical issues are likely to impact the outcome of MSC immunotherapy in different pathological contexts and explain some of the discrepancies found in clinical trials^14^. In addition to MSC features likely to influence their functional activity, such as donor variance, epigenetic reprogramming and senescence, alloimmunogenicity and the impact of cryopreservation, the discordance between the *in vitro* and *in vivo* suppressive effects ought to be considered, as reported^31^.

In addition to reporting novel molecules/mechanisms potentially involved in human AdMSC immunoregulatory activity, such as upregulation of MMP9 and PD-L1, in AdMSC, and of MMP2, BCL2 and GARP in T cells, we here show for the first time, that AdMSC with high suppressive activity display and induce a differential immunomolecular profile. Only in conditions where high suppressive activity was detected, we found specific correlations between increases in gene expression, connecting multiple immunoregulatory molecules, including MMP9, together with early simultaneous inhibition of IFN-γ, TNF-α, and IL-10 increase in the supernatant. Network analysis of gene expression modifications during AdMSC/PBMC interactions, showed MMP9 as a major node molecule during AdMSC suppressive activity. Upregulation of MMP9 and 2 was also confirmed at the protein level, in AdMSC and T cells, respectively, upon ongoing AdMSC suppressive activity. The inhibition of MMP2/9 lead to a significant decrease in AdMSC suppressive activity, confirming their important role. We conclude that MMP9 plays critical roles in MSC high suppressive potency, which also involves the capacity to induce and integrated multiple immunoregulatory mechanisms.

## Results

### Human AdMSC from different individuals have high and low suppressive capacity over T cell proliferation

**Figure 1:**
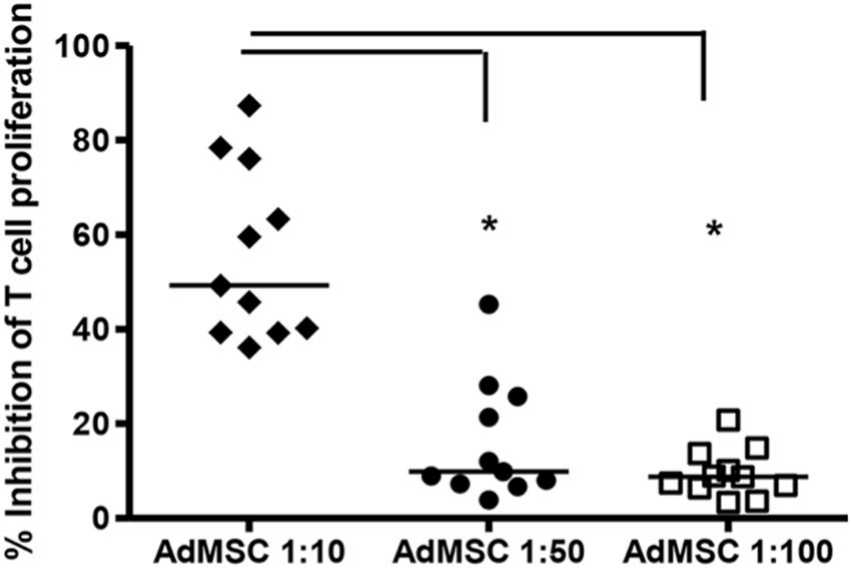
AdMSC immunosuppressive activity. AdMSC immunosuppressive activity over T cell proliferation induced by anti-CD3 monoclonal antibody after 5 days of culture. AdMSC were cocultured with different concentrations with CFSE-labeled PBMC stimulated with anti-CD3 antibody. AdMSC:PBMC ratios were 1:10, 1:50 and 1:100 (n = 11). After 5 days, PBMC were intracellularly labeled with anti-CD3 PE-CY5 antibody (Becton, Dickinson and Company, USA) and analyzed by FACS (FACScalibur, BD Company, USA), to determine AdMSC suppressive activity over the T cell proliferation, in a cell-cell contact condition. To calculate the percentage of proliferation inhibition, the following formula was used, considering all conditions with anti-CD3 stimulus: [(% CD3 + CFSE^low^ cells- % CD3+ CFSE^low^ cells with AdMSC)/% CD3+ CFSE^low^ cells] * 100. The background of CFSE-positive unstimulated cells was lower than 1%. Note assays displaying high (>50%) or low (<50%) suppressive activity. Percentage of inhibition of T cell proliferation was compared usind AdMSC:PBMC different ratios. One-Way ANOVA. *p < 0.05.

We evaluated AdMSC immunoregulatory activity over T cell proliferation in PBMC labeled with CFSE, stimulated with anti-CD3, *in vitro* (Supplementary Figure S1). All MSC (n = 11 individuals) inhibited T cell proliferation in a dose dependent manner (Fig. 1), using PBMC derived from a single individual and time point. AdMSC were more suppressive at 1:10 AdMSC/PBMC ratio (p < 0.05); the magnitude of inhibition ranged from 36 to 87% (Fig. 1). We classified AdMSC bearing high (>50% inhibition) or low (<50%) suppressive activity over T cell proliferation. Randomly repeated experiments confirmed consistent classification of AdMSC with high or low suppressive capacity (data not shown). We found no association between the magnitude of suppressive activity and the expression of molecules showing variable expression (HLA-I, CD44, CD49, CD73), used to characterize AdMSC (Supplementary Figure 2A and B).

### AdMSC/PBMC interactions increase/induce CD73^+^Treg subpopulation and decrease activated/effector T cells

We evaluated the effect of AdMSC/PBMC interactions, following anti-CD3 stimulation, on the expression of immune-related proteins, in AdMSC and T cells (n = 6 experiments). For T cells, we evaluated the percentage of different Treg (CD4^+^CD25^hi or low^FOXP3+) subpopulations, expressing CD73, CXCR3, CCR4, CCR5 and/or CCR7^34^. We observed increased percentage of the CD73^+^Treg subpopulation (CD4^+^CD25^hi^FOXP3^+^CD73^+^) at days 5 (p = 0.0313) and 8 of co-culture. (Supplementary Figure S3A and B). No change in other Treg subpopulations, CXCR3^+^Treg (Th1 Treg: CD4^+^CD25^hi^FOXP3^+^CXCR3^+^) and CCR7^+^Treg (Th2 Treg: CD4^+^CD25^hi^FOXP3^+^CCR7^+^) was observed (data not shown). AdMSC/PBMC interactions also decreased the percentage of CD8^+^ICOS^+^ (p = 0.0313), CD4^+^ICOS^+^ (p = 0.0156) and activated/effector T cells CD8^+^OX40^+^ T cells (Supplementary Figure S3C,D and E), especially for AdMSC displaying high suppressive capacity. In AdMSC, we observed increased percentages of CD90^+^CXCL10^+^CCL5^+^ cells (p = 0.0156) and CD90^+^PD-L1^+^ (p = 0.0156) (Supplementary Figure S3F and G).

### AdMSC with high suppressive capacity induce early and simultaneous decrease in TNF-α and IFN-γ in supernatants

**Figure 2:**
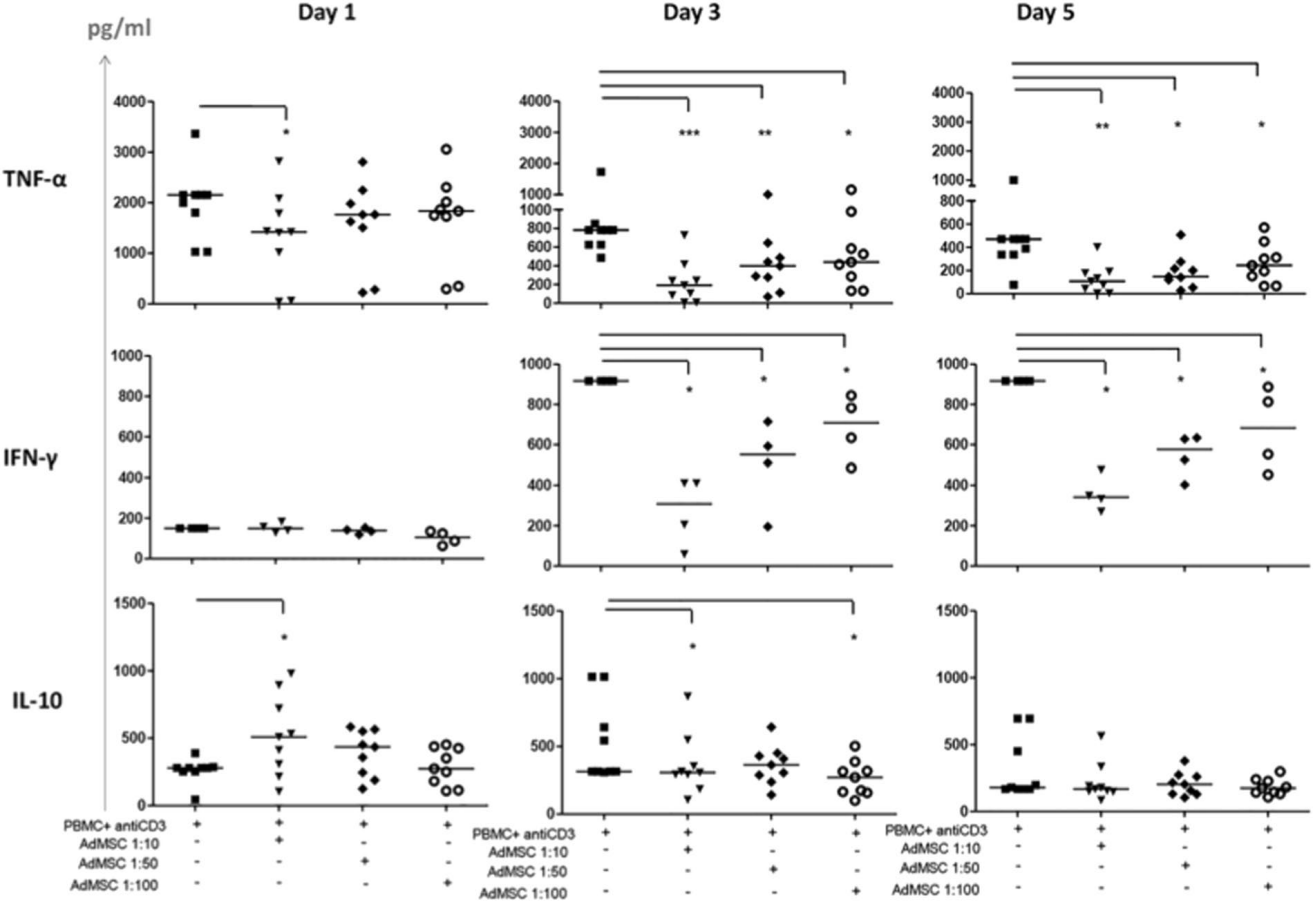
Effect of AdMSC on cytokine production in cocultures of PBMC stimulated with anti-CD3 antibody. Production of cytokines TNF-α, IFN-γ and IL-10 (pg/ml) in the supernatant of cocultures of AdMSC + PBMC at AdMSC: PBMC ratio (1:10, 1:50 and 1:100) after 1, 3 and 5 days of culture, measured by kit “Human Th1/Th2 CBA” (Cytometric bead Array) and analyzed by flow cytometry (n = 9 experiments with AdMSC derived from different individuals). AdMSC/PBMC interactions decreased TNF-α at all time points; induced an increase of IL-10 at day 1, and a decreased of IFN γ at days 3 and 5 (**A**). Detection limits of 5 pg/ml 5000 pg/ml. Mann Whitney test *p < 0.05, **p < 0.001. Only in assays with highly suppressive AdMSC we observed a positive correlation between the inhibition of TNF-α, and IFN-γ at day one of coculture (Correlation coefficient, ρ = 1.000, p = 0.0000) (B). For the assays displaying low suppression, the positive correlation between IFN-γ and TNF-α decrease appeared later, at days 3 (ρ = 0.8627, p = 0.0270) and 5 (ρ = 0.9963, p = 0.0000) (data no shown).

AdMSC/PBMC interactions increased IL-10 in the supernatant at day one of cocultures, and decreased IFN-γ and TNF-α at all time points (days 1, 3, 5 of culture) (Fig. 2A), but no significant changes for the other cytokines (data not shown). Cytokine kinetics allowed us to detect positive correlations among cytokine production changes during immunoregulation. Although IFN-γ and TNF-α decreased in assays with AdMSC displaying high or low suppressive capacity, only in the highly suppressive group we observed a positive correlation between the inhibition of these two cytokines at day one of coculture (Correlation coefficient, ρ = 1.000, p = 0.0000) (Fig. 2B **)**. For the assays displaying low suppression, the positive correlation between IFN-γ and TNF-α decrease appeared later, at days 3 (ρ = 0.8627, p = 0.0270) and 5 (ρ = 0.9963, p = 0.0000) (data not shown).

### AdMSC/PBMC interactions induce a dominant regulatory profile of gene expression modifications

**Figure 3:**
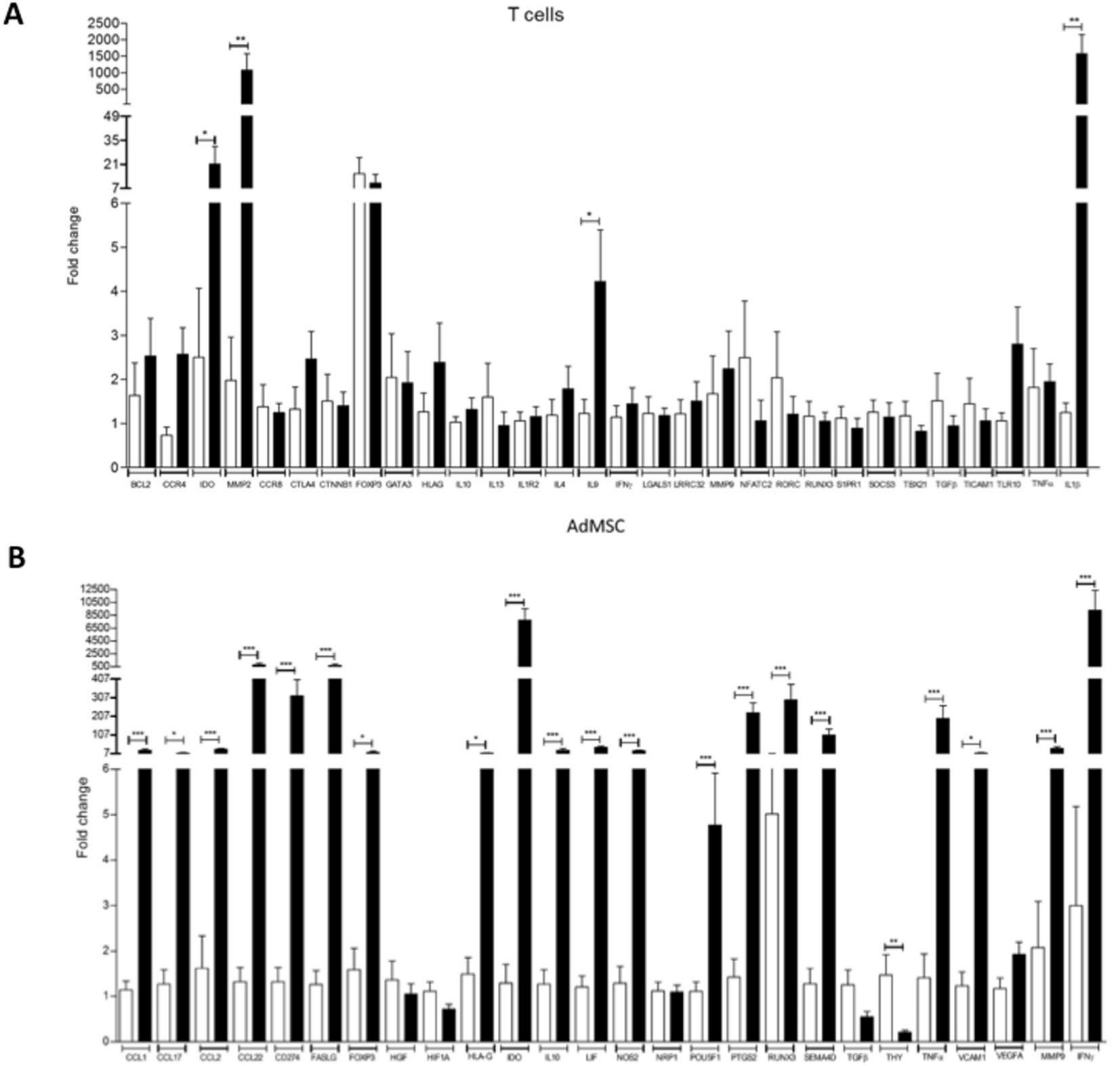
Effect of AdMSC/PBMC interactions upon gene expression in T cells and AdMSC. The relative expression of 30 genes were analyzed on magnetically purified T cells (**A**) and AdMSC (**B**) after 3 days of PBMC/AdMSC cocultures, stimulated with anti-CD3 antibody. The graph shows the fold change for the 30 genes analysed for PBMC/AdMSC cocultures (black bars) over the expression of magnetically purified cells cultured alone (white bars) with anti-CD3 stimulus (control). Statistically significant gene expression changes in relation to the control condition of cells cultured alone (p < 0.05) are marked shown *p < 0.05; **p < 0.01, ***p < 0.001. GAPDH was used as endogenous control for normalization. All experiments were performed with PBMC from a single individual. AdMSC and T cells (n = 8). Genes were classified by their predominant immunologic activity: immunoregulatory (REG), proinflammatory (INFLAMMA) and other predominant activities (OTHER). We calculated the REG/INFLAMMA ratios for gene expression changes upon AdMSC/PBMC interactions, during ongoing AdMSC suppressive activity. We considered REG gene expression changes: increase of predominantly REG genes or decrease of predominantly INFLAMMA genes; INFLAMMA gene expression changes: the opposite. REG/INFLAMMA ratios were: for T cell = 5.50; for AdMSC ratio = 2.72.

We evaluated gene expression changes of several immune-relevant molecules, during ongoing suppressive activity, *in vitro*, in AdMSC and T cells, and checked for differential profile regarding the magnitude of AdMSC suppressive activity. Both AdMSC and T cells displayed robust gene expression modifications during T cell proliferation inhibition. T cells presented upregulation of 11 molecules displaying predominantly immunoregulatory (REG) activity: IL4, CTLA4, FOXP3, IDO, MMP9, BCL2, TLR10, SOCS3, GARP, MMP2, and IL9, and one predominantly proinflammatory (INFLAMMA) molecule: IL1B (Fig. 3A). Three of these were dominantly upregulated (in all experiments) in T cells: IL1B, MMP2 and IDO.

In contrast, AdMSC showed significant mRNA upregulation for 21 molecules **(**Fig. 3B), some predominantly involved in chemoattraction (CCL1, CCL17, CCL22), migration (MMP9), immunoregulation (SEMA4D, CD274, FOXP3, HLAG, IDO, VCAM1, FASL, MMP9) and proinflammatory responses (IFNG, TNF, RUNX3) (Fig. 3B). Nineteen of these molecules were dominantly upregulated, in all experiments. IDO and IFNG were markedly upregulated (Fig. 3B).

The overall functional profile of gene expression modifications was predominantly regulatory for both T cells (Regulatory/Inflammatory gene expression modification ratio: 5.50) and AdMSC (Regulatory/Inflammatory gene expression modification ratio: 2.72). We detected both upregulation of predominantly REG genes and downregulation of predominantly INFLAMMA genes.

### MMP9 is a central node in specific networks integrating genes with upregulated expression in AdMSC and T cells during suppression

**Figure 4:**
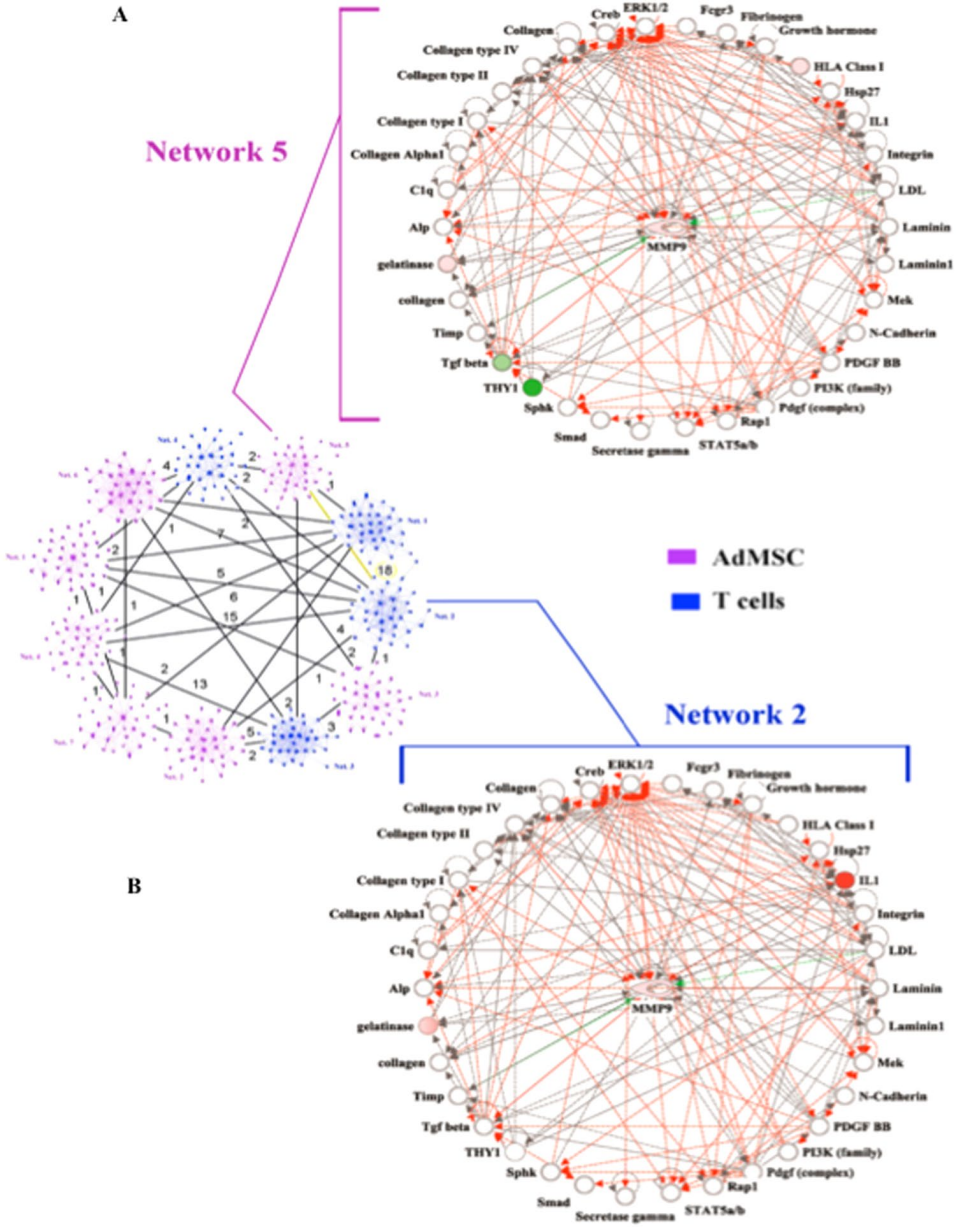
*In silico* analysis using the IPA software (Ingenuity Systems, Inc). Overlapping networks built with gene expression data from AdMSC (**A**) (purple) and T cells (**B**) (blue). The numbers represent genes or nodes in common between two networks. Highlighted in yellow the two networks (Number 5 for AdMSC and 2 for T cells) with greater number of nodes in common (18 nodes). The analysis shows two representative networks of molecules that include the genes differentially expressed in T cells (Network 2) and MSC (Network 5). Nodes are displayed using various shapes that represent the functional class of the gene product. The genes from the gene expression lists (focus molecules) are represented in graduation of red and green based on their fold change in expression. The white open nodes indicate proteins not identified in this analysis, but associated with the regulation of some of the genes analyzed. The green edges represent inhibition and red, activation. The networks were represented in a radial layout, placing the most connected node(s) in the center and arranging all other nodes around the rim of the circle. MMP9 is as central node in both networks.

We used IPA to identify molecular networks among differentially expressed genes in AdMSC and T cells during suppression. The software built 4 networks for T cells and 7 for AdMSC. We summarize the networks for AdMSC (Fig. 4A) and T cells (Fig. 4B); with score ≥ 2 or p ≤ 0.01 they overlap and share various genes. The networks 2 and 5 displayed higher number of common molecules, 18, (Network 2: AdMSC data; Network 5: T cell data; Fig. 4). The metalloproteinase 9 (MMP9) appeared as a central node, for both AdMSC (Fig. 4A) and T cells (Fig. 4B), when the networks were represented in a radial layout, placing the most connected node(s) in the center.

### Protein Upregulation of MMP9 MMP2 upon ongoing AdMSC suppressive activity

We determined the expression of MMP2, MMP9, in AdMSC, and T cells, at the protein level, upon ongoing suppressive activity, for some assays, comparing the basal expression in AdMSC and T cells alone and following AdMSC – PBMC interactions, during suppressive activity.

**Figure 5:**
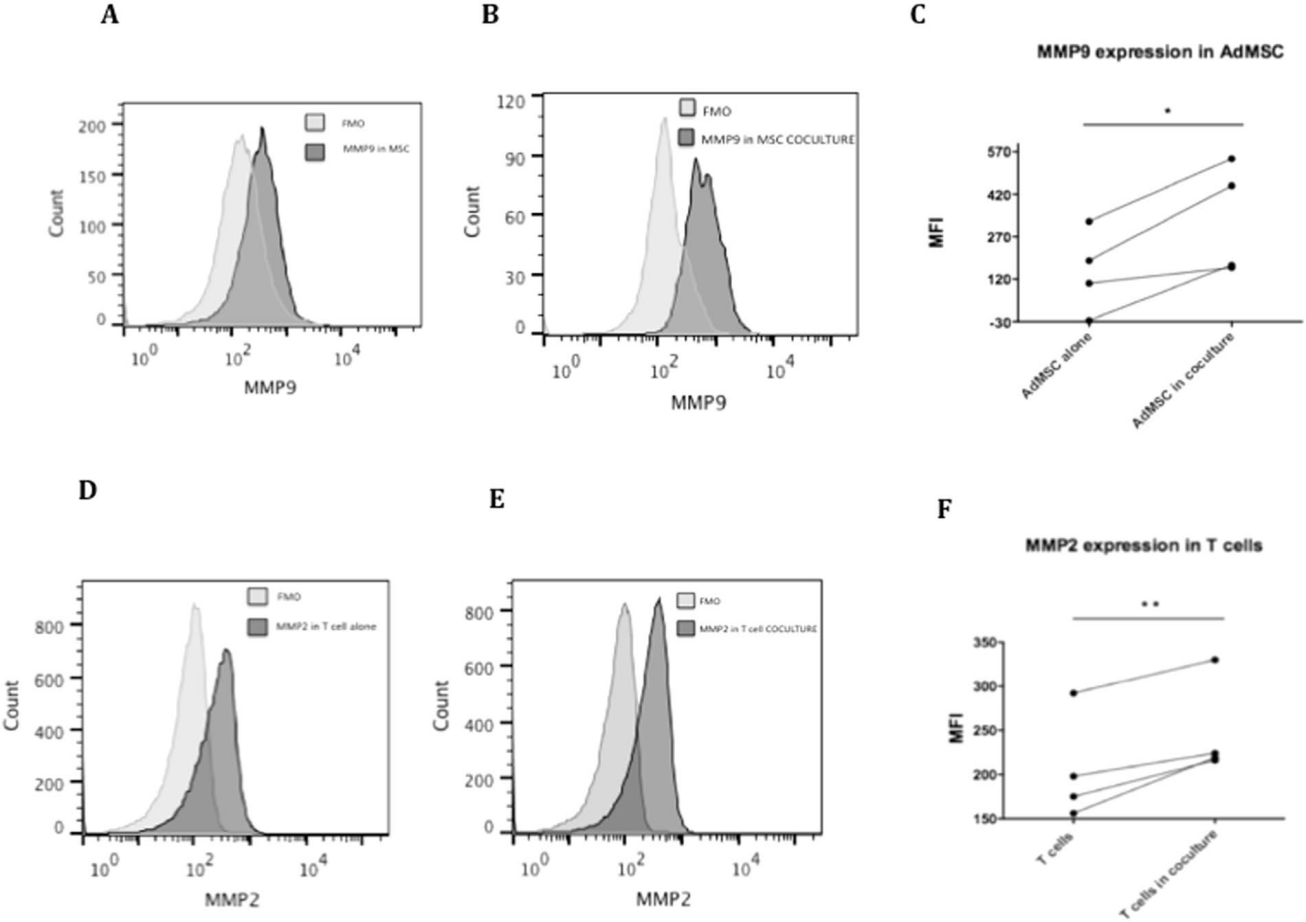
Protein expression of MMP9 in AdMSC and MMP2 in T cells. The median protein expression of MMP9 in AdMSC alone (**A**) and in AdMSC in coculture with PBMC (**B**) compared to corresponding FMO. Increase in the expression of MMP9 in AdMSC following coculture, during AdMSC supressive activity (n = 4) (**C**). The median protein expression of MMP2 in T cells alone (**D**) and in T cells in coculture with AdMSC (**E**) compared to corresponding FMO. The protein expression of MMP9 was analyzed in AdMSC alone and AdMSC in coculture with PBMC; MMP2 in T cells alone and in T cells in coculture with AdMSC after 3 days of culture, stimulated with anti-CD3 antibody (**F**). After culture cells were stained with anti-MMP9 FITC and anti-MMP2 PE antibody analyzed by flow cytometry using the DIVA program for acquisition and Flow Jo for analysis. A total of 100,000 events were analyzed within the lymphocyte gate selected by size (FSC) and granularity (SSC). Paired t test *p = 0.01 (MMP9) and **p = 0.006 (MMP2).

In concordance with the gene expression data, we found significant protein expression upregulation of MMP9 (Fig. 5A and B; p = 0.01, paired t-test for median fluoresecence intensity – MFI and p = 0.03, paired t-test for fold increase) for AdMSC, and of MMP2 (Fig. 5C and D; p = 0.006, paired t-test for median fluoresecence intensity – MFI and p = 0.03, paired t-test for fold increase) for T cells. The increase of MMP9 did not reach statistical significance for T cells (p = 0.05, paired t-test for median fluoresecence intensity – MFI) nor did the increase of MMP2 for AdMSC (p = 0.07, paired t-test for the percentage of positive AdMSC).

In addition, also in concordance with our gene expression data, we found significant upregulation of PD1L (p = 0.0008, paired t-test for median fluoresecence intensity – MFI) on AdMSC (Suplementary Fig. 3J), and an increase in the percentage of CCR4 (p = 0.04, paired t-test) and CTLA4 (p = 0.007, paired t-test) (Suplementary Fig. 4) positive T cells, at the protein level.

### MMP2/9 are important new players in human AdMSC immunoregulatory

We selected four molecules whose expression dominantly increased during AdMSC suppressive activity (MMP2/MMP9, CD73, HLAG, IDO), to further evaluate their participation and potential synergy/interactions in immunosuppressive mechanisms over T cell proliferation, using their inhibitors.

**Figure 6:**
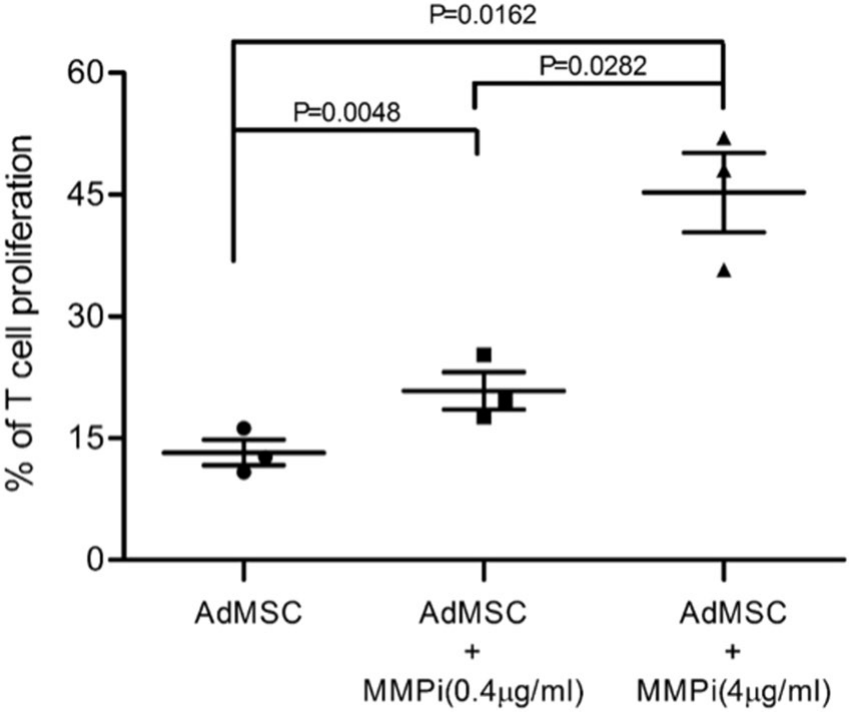
MMP2/9 are major players in human AdMSC immunoregulatory mechanisms and suppressive potency. The significant decrease in AdMSC suppressive capacity in inhibition experiments confirm that MMP2/9 are major players in the suppressive potency of human AdMSC. CSFE labeled PBMC, stimulated with anti-CD3 antibody (1 μg/ml), were cultured alone or with AdMSC, for 5days, with or without the MMP2 and MMP9 inhibitor - SB-3CT (0.4 ug/ml and 4 ug/ml). The cells were acquired in FACS CantoII flow cytometer using the program DIVA for acquisition and Flow Jo for analysis. A total of 300,000 events were analyzed within the lymphocyte gate selected by size (FSC) and granularity (SSC). Paired t test: p = 0.0048 (0.4 ug/ml), p = 0.0162 (4 ug/ml).

All inhibitors partially reestablished T cell proliferation in a dose dependent manner, indicating a negative impact on AdMSC’s immunoregulatory effect (Fig. 6). However, none of the inhibitors used alone abolished AdMSC suppressive activity. In line with the IPA analysis, MMP2/9 inhibitor significantly restored T cell proliferation (Fig. 6), indicating that MMP9 and MMP2 have a central role in human AdMSC immunoregulatory mechanisms and their high suppression potency. We combined iMMP with iIDO, iCD73 and iHLAG to test synergic action further decreasing AdMSC suppressive activity, but found no additional impact (data not shown).

### Highly suppressive AdMSC display/induce a differential immunomolecular profile indicating interplay of multiple immune molecules and mechanisms

**Figure 7:**
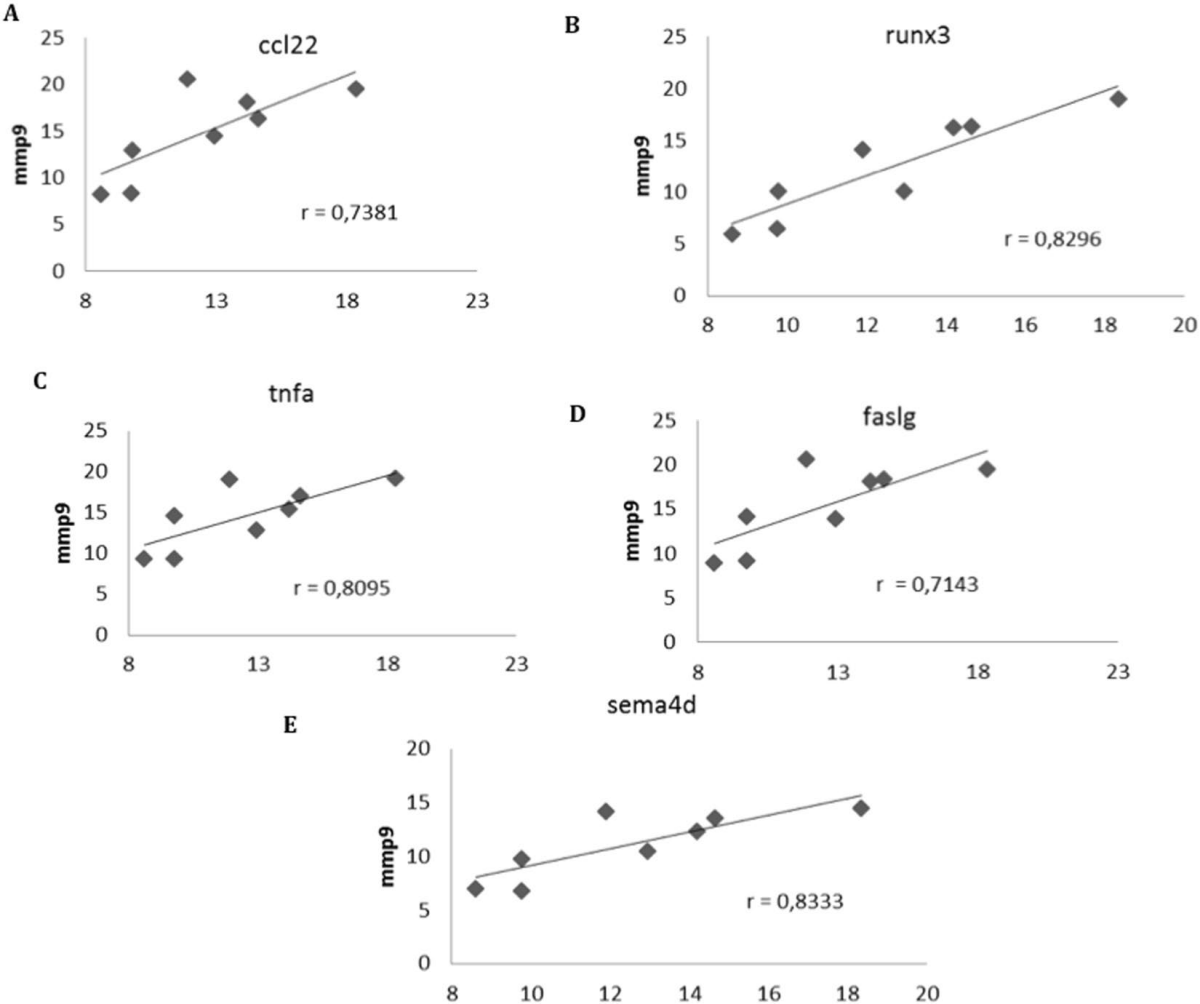
Specific Positive correlations of MMP9 gene expression upregulation in AdMSC, exclusively found in the assays displaying high immunosuppressive activity. The increase of MMP9 was positively correlated with the increase of CCL22 (**A**) (p < 0.05), RUNX3 (**B**) (p < 0.001), TNF (**C**) (p < 0.05), FASL (**D**) (p < 0.05) and SEMAD4 (**E**) (p < 0.05), only in highly suppressive AdMSC. Several other positive exclusive correlations among increases in gene expressions were found in assays with high inhibition of T cell proliferation (>50%). The correlation coefficients are shown in graphs, using Spearman correlation test. Definitions of all abbreviations are discriminated on Supplemental Table S1.

**Figure 8:**
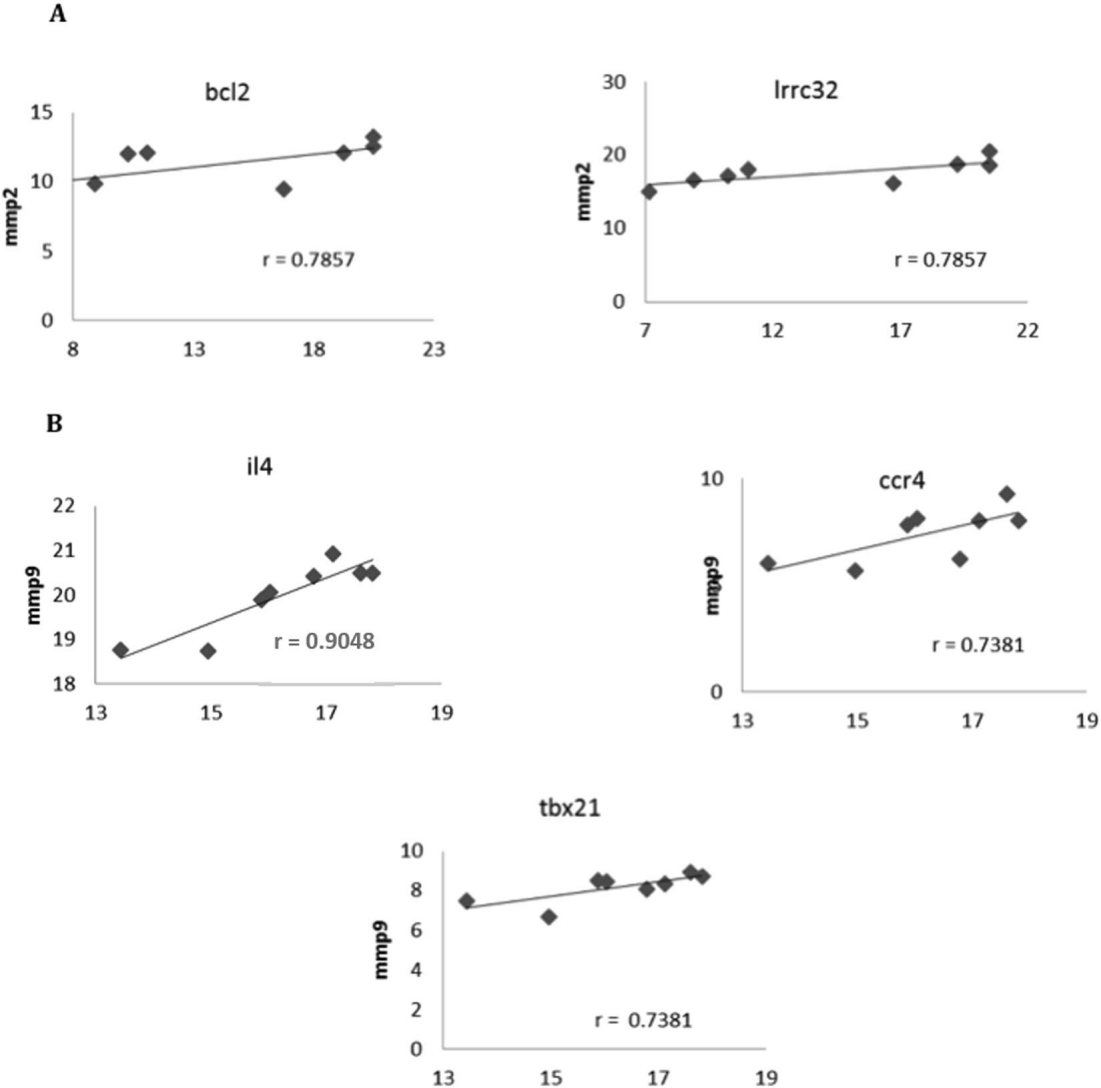
Specific Positive correlations of MMP2 and MMP9 gene expression upregulation in T cells, exclusively found in the assays displaying high immunosuppressive activity. The increase of MMP2 (**A**) was positively correlated with the increase of BCL2 (p < 0.05), LRRC32 (p < 0.05) in T cells. The increase of MMP9 (**B**) was positively correlated with the increase of IL4 (p < 0.01), CCR4 (p < 0.05) and TBX21 (p < 0.05) in T cells. Several other positive exclusive correlations among increases in gene expressions were found in assays with high inhibition of T cell proliferation (>50%). The correlation coefficients are shown in graphs, using Spearman correlation test. Definitions of all abbreviations are discriminated on Supplemental Table S2.

We found various specific correlations of gene expression modifications, only in cocultures displaying high (>50% proliferation inhibition) immunosuppressive activity (Hi-Sup), for both AdMSC and T cells. We highlight some of these specific positive correlations in Hi-Sup assay, such as between the increase in gene expression of MMP9 and CCL22 (Fig. 7A), RUNX3 (Fig. 7B), TNF (Fig. 7C), FASL (Fig. 7D) and SEMAD4D (Fig. 7E) in AdMSC. For T cells, we found specifc positive correlations between the increase in gene expression of MMP2 (Fig. 8A) and BCL2 and LRRC32, and also between the increase of MMP9 (Fig. 8B), and IL-4 and CCR4 and TBX21. Only in Hi-Sup assays, we found any correlation between increased mRNA expression of important immunoregulatory molecules, namely, IL4, GATA3, HLAG, FOXP3, IDO, BCL2, CCR8 and IL10 in T cells. Likewise, in AdMSC, only in Hi-Sup assays, we detected correlations between increased mRNA expression of CCL22, IL-13 and TNF.

We used IPA to build interaction pathways of the genes whose upregulation occurred in a correlated manner, exclusively in Hi-Sup assays. Again, MMP9 appears as a nodal molecule in both networks, directly or indirectly connecting most molecules with high correlation to IL4 and TNF in the T cells and AdMSCs networks, respectively.

**Figure 9:**
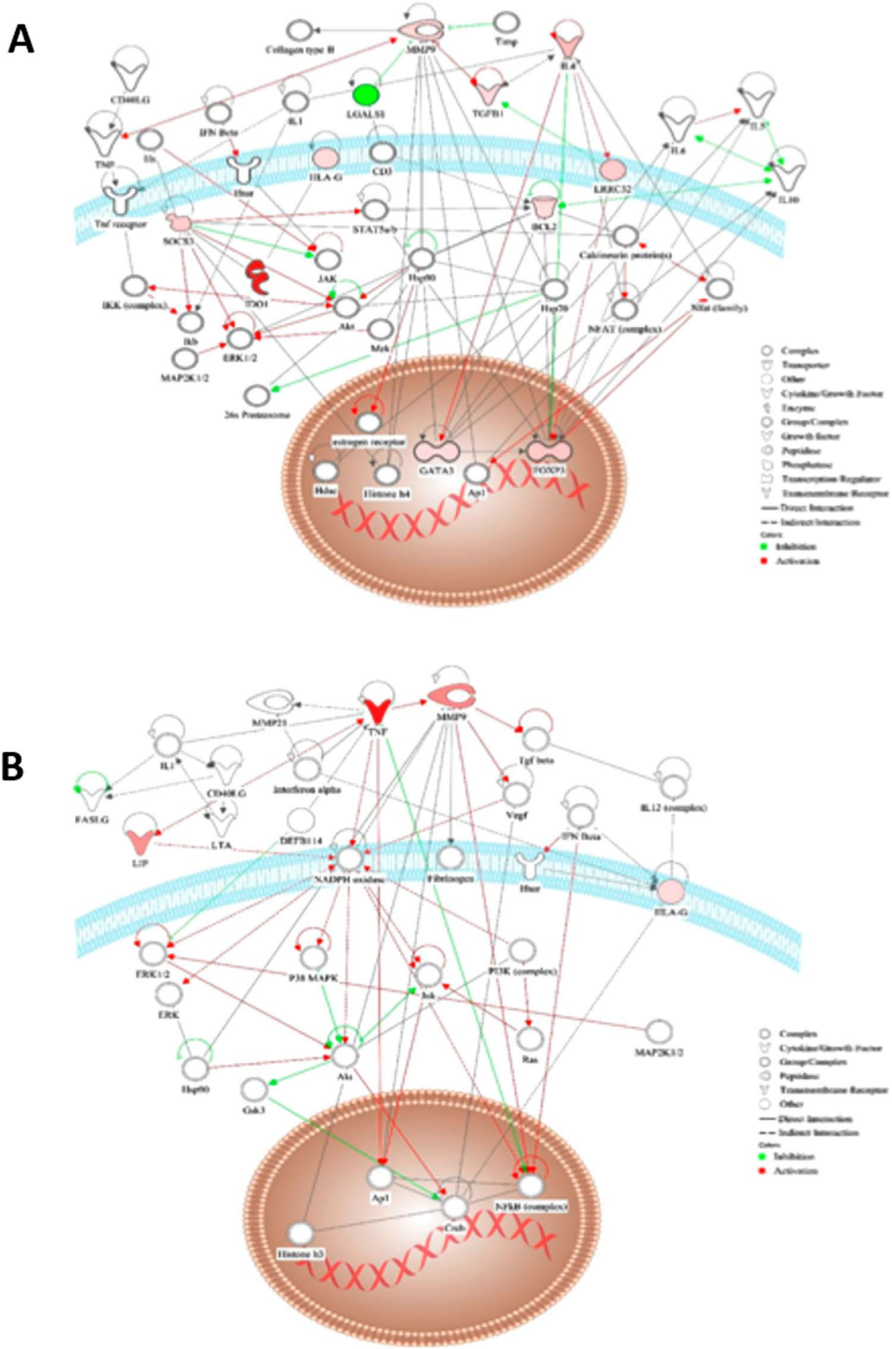
Network for T cells and AdMSC correlated gene upregulation in assays with high suppressive activity. (**A**) Representative network built, using IPA software, for the genes or “focus molecules” that presented positive correlation in gene expression with IL-4 in T cells, only in the assays with highly suppressive AdMSC. (MMP9, LGALS1, LRRC31, SOCS3, TGFB1, BCL2, CCR8, FOXP3, GATA, HLAG and IDO). (**B**) Representative network built, using IPA software, for the genes or “focus molecules” that presented positive correlation in gene expression with TNF in AdMSC, only in the assays in which AdMSC presented high suppressive activity. (MMP9, HLAG and LIF). The network is represented in a subcellular layout, placing the nodes into a view of subcellular compartments. The genes are represented in graduation of red and green based on their fold change in expression. The white open nodes indicate molecules not identified in this analysis, but associated with the regulation of some of the genes analyzed. The green edges represent inhibition and red, activation.

We show two networks for the genes with exclusive positive correlations with TNF in AdMSC (MMP9, HLAG and LIF) (Fig. 9A) and with IL4 in T cells (MMP9, LGALS1, LRRC31, SOCS3, TGFB1, BCL2, CCR8, FOXP3, GATA3, HLAG and IDO) (Fig. 9B), representing subcellular and spatial location of molecules. For T cells, the network (Fig. 9B) shows functions related to “Cellular Movement, Hematological System Development and Function, Immune cell trafficking and cell-mediated immune response”, with a highly significant score of 12 (p = 10^−12^) (Fig. 9). In the T cell network, IL4 appears directly connected with IL1-complex and indirectly to TGFB1 and with the cytoplasmic complex NFat (Nuclear factor of activated T cells). In the nucleus, the transcription factors GATA-3 and FOXP3 are also directly connected to IL-4.

For AdMSCs, the network (Fig. 9A) shows functions related to “Nervous System Development and function, tissue morphology and inflammatory response”, with a highly significant score of 12 (p = 10^−12^). In the AdMSC network, TNF is directly connected to IL1-complex and MMP9 in the extracellular space, and to the transcription regulators complexes: NFκB and AP1 in the nucleus. In the plasma membrane, TNF indirectly connects to the NADPH oxidase complex, which is a nodal molecule connected to a great number of molecules, with a central role in the network.

## Discussion

Despite current discussion on MSC phenotypic/functional heterogeneity^14, 37, 38^, little is known about the functional relationship among the various molecules/mechanisms involved in MSC activity and the intensity of MSC suppressive capacity, especially in human MSC. We have started tackling these questions, by evaluating the effect of AdMSC activity on the expression of various immune-relevant molecules, during AdMSC/PBMC interactions and ongoing suppressive activity, in both AdMSC and T cells, and the impact on cytokine production and on various effector and regulatory immune cell subpopulations, comparing AdMSC’s high or low suppressive capacity over T cell proliferation.

We here show that AdMSC suppressive activity, *in vitro*, involves the simultaneous activation/mobilization of multiple molecules in both AdMSC and T cells, indicating that multiple molecular pathways act simultaneously. AdMSC with high suppressive activity is determined by the capacity to mobilize a differential set of multiple imune-related molecular pathways, in a correlated manner. Interestingly, various important molecules involved in immunoregulation or cell survival, such as IL-4, FOXP3, GATA-3, IL-13, IL-10 and BCL2, only presented upregulation in a correlated manner in assays with highly suppressive AdMSC. Within this complex network, we found that MMP9 is a robust new player involved in the molecular network of MSC’s high suppressive capacity. In addition, we show that MSC with high suppressive capacity induce an increase of IL-10 and early inhibition of IFN-γ and TNF-α production, in a correlated manner, not found in assays with low suppressive activity.

Some gene expression changes were strikingly dominant - occurring in all experiments - such as, increased IDO in T cells and AdMSC, and IL1β and MMP2 in T cells. In fact, nineteen out of the 30 genes studied for AdMSC displayed a dominant upregulation, including MMP9, HLA-G, IL10, PDL1, SEMAD4, FASL, RUNX3, TNF, IFNg and several chemokines. Although IDO^20^, HLA-G^23^, PDL1^25, 39^ and IL10^22^ have been previously reported as relevant to AdMSC suppressive activity, it was not known that they are simultaneously and dominantly mobilized. In line with the highlighted importance of TFNα increase for MSC suppressive activity, inducing NFkB activation^40^, we found a dominant TNF mRNA increase in AdMSC, in all experiments. Although the upregulation of TNF in AdMSC, itself, was unrelated to AdMSC immunosuppressive intensity, correlated upregulation was only found in assays with highly suppressive AdMSC, namely with HLAG, LIF and MMP9.

The IPA analysis using all differentially expressed genes in AdMSC and T cells, showed MMP9 as a central node during AdMSC suppressive activity, in both networks, for T cells and AdMSC. Since central node molecules have been shown to play a crucial role in connecting the molecular network^35, 36^, our IPA analysis points MMP9 as an important player in AdMSC suppressive activity. Moreover, MMP9 appeared as an integrating molecule in the networks built with the genes displaying positive correlation with the upregulation of IL4 in T cells and TNF in AdMSC, exclusively in assays with high suppressive activity. The significant upregulation of MMP9 expression during AdMSC suppressive acitivity, was also confirmed at the protein level.

We tested whether MMP9 and other dominantly upregulated molecules (IDO, HLAG), played a critical role in AdMSC suppressive activity. The inhibition of these molecules, *in vitro*, decreased but did not abolish AdMSC suppressive activity, indicating immunoregulatory activity occurring in concert with other players. The inhibition of MMP9, indeed, significantly decreased AdMSC high suppressive activity, supporting its relevant participation in immunoregulatory mechanisms and in suppressive potency. TNF, dominantly upregulated in AdMSC, has a direct relationship with MMP9, increasing MMP9 enzymatic activity^41^, and this MMP9-TNF correlated upregulation was only found in highly suppressive AdMSC, suggesting the need of an integrated action of these molecules for higher suppressive activity. MMP9 upregulation also correlated with the increase of CCL22, exclusively in highly suppressive AdMSC, suggesting favorable conditions for Treg recruitment. In the IL4 network build for T cells, we found a direct relationship of IL4 with TGF and TGF with MMP9. IL-4 increases TGF, which alone increases MMP9 activity^42^. Again, only in assays with highly suppressive AdMSC, we found correlated upregulation of IL-4, in T cells, with several molecules, including with MMP9, FOXP3 and GATA-3.

Noteworthy, MMP2 is also affected by the inhibitor used and also integrates our IPA networks. We confirmed upregulation of MMP2 at the protein level, in T cells, upon AdMSC/PBMC interactions. It is, therefore, likely that MMP2 also contributes to AdMSC high suppressive activity, through T cell activity. Only in assays with highly suppressive AdMSC, we found correlated upregulation of MMP2 and BCL2 and LRRC32 (GARP), in T cells, suggesting that mobilizing Tregs acting through TGF-beta and promoting cell survival are involved in AdMSC high suppressive activity. Moreover, the expression of GARP on human Tregs has been reported to be related to their higher suppressive acitivy. Although, both proinflammatory^43^ and immunoregulatory activities have been suggested for MMP2^44, 45^, immunoregulation seems predominant in the context of AdMSC activity. MMP9 has been implicated in murine bone marrow MSC suppressive activity, *in vitro* and *in vivo* ^46^, and reported in human bone marrow MSC as an important molecule for homing/migration into tissues, in response to inflammatory stimuli^47^. Moreover, bone marrow MSC prevented murine islet allograft rejection by MMP2/9 suppressive activity^46^. We now show the importance of MMP/9 as novel relevant and dominant participants in human AdMSC suppressive mechanisms and potency.

The increased numbers of Tregs expressing CD73, an ectoenzyme that induces adenosine - an immunosuppressive molecule^49^, indicates another MSC immunoregulatory mechanism. Accordingly, others have reported the induction of CD4^+^CD25^+^CD73^+^ T cells by human MSC^50^. We now show that the expansion of CD73^+^CD4^+^CD25^+^ Tregs during AdMSC immunoregulatory activity, actually occurs in cells with stable FOXP3 expression, characteristic of Tregs but not activated T cells. Using the CD73 inhibitor, we observed some decrease in AdMSC suppressive activity in individual experiments, but no statistical significance, either alone or in combination with IDO inhibitor. This suggests that, at least *in vitro*, the expression of CD73 by Tregs may not be a dominant feature in the AdMSC suppressive activity.

We also found decreased numbers of activated/effector CD4^+^ and CD8^+^ cells expressing ICOS, and CD8^+^ cells expressing OX40, molecules implicated in effector T cell costimulation and activation^51^, respectively. As ICOS requires *de novo* induction on T cell surface, being upregulated in a late phase of T cell activation^52^, our data suggest that AdMSC also act upon a late phase of T cell activation.

The increased TNFα, IFNγ and IL1B mRNA expression in AdMSC underscores the importance of proinflammatory cytokines, enhancing MSC immunosuppressive activity, as reported^26, 53^. Indeed, many proinflammatory molecules trigger a network of immunoregulatory molecules, as reported for IFN-γ inducing IDO and PD-L1^20, 39, 44^ in the context of MSC immunosuppression. In addition, IFN-γ along with TNF-α, IL-1α and IL-1β induces iNOS and CXCL10 and CXCL9, also important in cell trafficking in immunoregulation^30^. The marked mRNA upregulation of CCL22 and CCL17 and of CXCL10 and CCL5 at the protein level, in AdMSC during suppressive activity, suggest a favorable context for Treg migration and recruitment, as reported^30, 45^, corroborating these interpretations.

The magnitude of MSC suppressive activity and implicated mechanisms have been largely overlooked in the literature particularly in clinical trials. Considering the potential impact on immunodulatory cell therapy outcome, we believe it is crucial to determine whether high or low magnitude of MSC suppressive capacity provides the best beneficial therapeutic effect, in specific pathological contexts, and underlying mechanisms. The recent findings that T cells from autoimmune disease patients have reduced sensitivity to immunoregulation by MSC^54^ further highlights the importance of determining these issues for immunomodulation cell therapy in the clinic.

Besides adding novel players to the scenario of MSC immunobiology, our data bring a relevant contribution, providing a broader view of the interactive immunoregulatory molecular networks involved in MSC immunologic activities. The more potent MSC suppressive activity correlates to their capacity to trigger a coordinated action of multiple molecules, mobilizing various immunoregulatory mechanisms. We highlight MMP9 emerging as an integrating molecule in human AdMSC immunregulatory network and the importance of the early inhibition of proinflammatory cytokines, along with the induction/increase of IL-10, contributing to a more potent immunoregulatory capacity. Taken that MSC are present throughout the body, we may interpret that the multiple MSC interactions with a variety of immune cells - and other cell types - along evolution, have favored these stromal cells to develop an enormous variety of immunoregulatory mechanisms, likely to contribute, *in vivo*, to the maintenance of *in situ* and peripheral tolerance, and the control of homeostasis, supporting the idea that MSC may integrate the immune system.

## Material and Methods

### Experimental Design

To measure AdMSC suppressive capacity over T cell proliferation (anti-CD3 stimulated), we cocultured AdMSC (different individuals, n = 11) with PBMC from a single healthy individual, favoring the evaluation of AdMSC immunoregulatory variability individual-dependent. PBMC from an additional individual were used for some experiments. We classified AdMSC as displaying high (>50% inhibition) or low (<50%) suppressive activity. We evaluated several immunologic features following AdMSC/PBMC interactions and tested for correlations of gene/protein expression modifications considering high (>50% inhibition) or low (<50%) suppressive capacity. Features analysed: (i) T cell proliferation inhibition; and changes in (ii) gene expression of immune-relevant molecules in AdMSC and T cells; (iii) proinflammatory and immunoregulatory cytokine production; (iv) regulatory and effector T cell subpopulations. Using inhibition assays, we evaluated the contribution of selected molecules to AdMSC suppressive activity.

### Study Subjects

This study was approved by the local Ethics Committees (*Comissão de Ética para Análise de Projetos de Pesquisa do HCFMUSP*- 0765/09) and all methods were performed in accordance with the relevant guidelines and regulations (Comissão Científica da Fundação Pró-Sangue – 005/2009). Following informed consent, eleven healthy individuals donated AdMSC and two PBMC. Subject features: mean age of AdMSC donors: 40 years old (28–67 yrs); 3 males (2 for PBMC), 8 females; overweight: n = 5, obese: n = 3, normal weight: n = 1, (body mass index - BMI: underweight: <18.5; normal: >18.5 < 25; overweight: >25 < 30; obese: IMC > 30).

### AdMSC expansion and phenotypic characterization

Lipoaspiration (20 ml) AdMSC were obtained by collagenase IA-mediated (20 mg; Sigma-Aldrich, USA), digestion at 37 °C for 30 min, cultured for 3 days until 80% confluence, trypsinized and expanded. AdMSC immunophenotypical characterization (n = 11; passages 4–6) was done with: anti-: CD90FITC (Clone 5E10), HLA-ABCPE (Clone G46-2.6), CD49ePE (Clone IIA1), CD13PE (Clone WM15), CD73PE (Clone AD2), CD29PE (Clone MAR4), CD44 FITC (Clone G44-26), CD105PECY5 (Clone 266), CD106PE (Clone 51-10C9), CD14PE (Clone M5E2), CD31FITC (Clone WM59), CD51/61FITC (Clone 23C6), CD133PE (Clone AC133), CD45PE (Clone HI30), HLA-DRPE (Clone G46-6), CD34PE (Becton, Dickinson and Company, NJ, USA) using FACSCalibur (B&D, Becton, Dickinson and Company, NJ,USA). We analyzed 20,000 events by size (FSC) and granularity (SSC). All AdMSC were positive for CD90, CD13, CD73, CD49e, CD29, CD44, HLA-ABC; negative for CD34, CD45, CD51/61, CD133, HLA-DR. AdMSC expression of HLA-I, CD44, CD49 and CD73 varied. (Supplementary Figure S2). AdMSC culture for adipocyte differentiation: α-MEM (Gibco-BRL,USA), 15% FBS, 10 M dexamethasone (Decadron injectable Prodomo, Brazil), 10 mg/ml insulin (Sigma-Aldrich,USA), 100 mM indomethacin (Sigma-Aldrich, USA). Osteocyte differentiation: α-MEM (Gibco-BRL, USA), 7.5% FBS, 10 M dexamethasone (Decadron injectable Prodomo, Brazil), 100 M ascorbic acid, 10 mM β glicerolphosphate (React, Brazil). For cell morphology analysis post-differentiation: von Kossa method (for calcium deposition: osteocyte) (Supplementary Figure S2), Oil Red (for lipid accumulation: adipocyte) (Supplementary Figure S2).

### PBMC Isolation

PBMC were isolated from aphaeresis leukocyte filters by Ficoll **(**Ficoll: Pharmacia Biotech, Sweden and Hypaque: Schering, Brazil) and cryopreserved in FBS/DMSO 90%/10%, and liquid nitrogen, until use. Cell viability ≥85%.

### Proliferation Assays to evaluate AdMSC suppressive activity

PBMC proliferation was assessed by FACS using Carboxyfluorescein Succinimidyl Ester (CFSE). PBMC (1 × 10^6^ cells/ml) were incutated with CFSE (2.5 µM) at 37 °C, 10 min; staining quenched with ice-cold 10% FBS (Hyclone, USA) RPMI (Gibco, USA). AdMSCs were plated (1 × 10^5^); after 24 h, CFSE-labeled PBMC were added (1:10, 1:50, 1:100 AdMSC/PBMC ratios). Cells were cultured in 10% FBS RPMI (Sigma-Aldrich, USA) and anti-CD3 (1 µg/ml; OKT3; Janssen-Cilag BV, The Netherlands). Baseline control: unstimulated PBMC. After 5 days, PBMC were intracellularly labeled with anti-CD3PE-CY5 (Becton, Dickinson and Company, USA), analyzed (FACScalibur, BD Company, USA) to determine AdMSC suppressive activity over T cell proliferation, (cell-cell contact). Proliferation inhibition was calculated as: [(% CD3^+^CFSE^low^cells-% CD3^+^CFSE^low^cells with AdMSC)/% CD3^+^CFSE^low^cells]*100 (all conditions stimulated with anti-CD3). CD3^+^CFSE^low^ background of unstimulated cells: <1%. (Supplementary Figure S1).

### AdMSC/PBMC Coculture Experiments for RNA extraction

AdMSC/PBMC were cocultured for 3 days as in proliferation assays. PBMC were collected and adherent cells detached (Triple Select; Gibco, USA). For AdMSC and T cell purification, we first collected the cells that were in suspension from the co-culture (PBMC), washed and collected them again (Suspension A). Then, we washed adherent AdMSC with DPBS (Gibco, Rockville, USA), detached adherent them with Tryple Select (Gibco, Rockville, USA) and collected them separately (Suspension B). Then, we used the PanT cell kit (Miltenyi Biotec GmBH Bergisch Gladbach, Germany,) for magnetic negative selection of T cells (which contains the following antibodies anti- CD14, CD16, CD19, CD36, CD56, CD123 and CD235a) using suspensions A and B, separately. Therefore, all non-T cells such as monocytes, NK, B cells erythrocytes, granulocytes and dendritic cells remain attached to the column and, as T cells and MSC have none of these molecules, they do not adhere to the column. Then, to obtain T cells from Suspension A, we used the CD4+ and CD8+ kits for magnetic positive selection (Miltenyi Biotec GmBH Bergisch Gladbach, Germany). Cell purity: 70–90% (FACS). Isolated cells were stored in RNAprotect® Cell Reagent (Qiagen, AMBION,USA) at −80 °C, until RNA extraction.

### Isolation of total RNA and cDNA synthesis

Genomic DNA-free total RNA was isolated (RNeasy Plus Mini kit, Qiagen, AMBION, USA), following the manufactures’ protocol. RNA concentration and integrity were evaluated by NanoDrop-1000 (Thermo Scientific), UV/Vis ratios and agarose gel. For cDNA synthesis: 3 μg of total RNA; kit RT^2^ First Strand Kit (SuperArray Bioscience Corporation,USA).

### Analysis of mRNA expression by Real-time PCR

Real Time PCR was performed using customized 96-well plates (RT^2^ PCR Array custom Profiler; SuperArray Bioscience Corporation, USA), (30 genes for AdMSC, 30 for T cells) (Supplementary Tables S1 and S2). We used a Master Mix with SYBR Green (SuperArray Bioscience Corporation, USA) (final volume: 25 µl), the ABI Prism 7500 Real Time PCR System (Applied Biosystems), and GAPDH for normalization. Gene expression changes following PBMC/AdMSC interactions were calculated relative to PBMC or AdMSC alone. We considered increased or decreased mRNA expression when the relative expression (R.E.) was ≥2.0 and ≤0.5, respectively and statistically significant (P < 0.05, Wilcoxon). For gene expression quantification we used the SABiosciences software (http://www.sabiosciences.com/pcrarraydataanalysis.php). Undetectable: Ct >35. Gene expression changes were also characterized as exhibiting a dominant regulatory (REG: R.E ≥ 2 for regulatory genes or ≤0.5 for inflammatory genes) or an inflammatory (INFLAMMA: the opposite of REG) profile.

### Network analysis using Ingenuity Pathway Analysis (IPA)

IPA Network maintains a graphical database of molecular network interactions (Ingenuity Knowledge Base, IKB). We uploaded in the IPA all genes differentially expressed in AdMSC and T cells, following AdMSC/PBMC interactions, and genes with positive correlations in T cells and AdMSC, only in the assays with high suppressive activity. The genes were mapped to their corresponding gene objects in the IKB and overlaid onto a global network based on the IKB. Smaller networks (<35 nodes) containing a significant number of focus genes were algorithmically selected and scored; a score ≥ 2 indicated a probability p ≤ 0.01 that the focus genes in a network were found together by chance.

### Evaluation of AdMSC and Regulatory or Effector T cell subpopulations

We evaluated the effect of AdMSC/PBMC interactions under anti-CD3 stimulation, on the expression of various immune-related proteins in AdMSC and T lymphocytes, and quantified some Treg/effector subpopulations (FACS). AdMSC (3 × 10^5^) were cultured in 10% FBS αMEM medium (Gibco-BRL, USA) for 24 h, for adherence. PBMC (3 × 10^6^) were plated on AdMSC in 10% FBS RPMI, 1:10 AdMSC/PBMC ratio. Conditions: (i) PBMC+anti-CD3; (ii) PBMC+anti-CD3+AdMSC; (iii) AdMSC+anti-CD3. After 3 days, we analysed: AdMSC in CD90^+^ gated cells: % of PD-L1^+^, LAP^+^ (*latency-associated peptide*), HLADR^+^, CD80^+^, CD86^+^ and CXCL10^+^CCL5^+^; T cells: gated considering FSC/SSC: % of different Treg (CD4^+^CD25^hi or low^FOXP3^+^) subpopulations, expressing CD73^+^, CXCR3^+^, CCR4^+^, CCR5^+^ and CCR7^+^^34^, and the CD4^+^CD25^hi^ FOXP3^+^CD73^+^ Treg subpopulation at coculture day 8. In this case, the cells were fed at day 4, by changing culture medium supplemented with IL-2 (20 U/ml). We also evaluated: % of ICOS (*inducible co-stimulator*) and OX40 *(or CD134, TNFR family member)* on CD4^+^ and CD8^+^ T cells, and the median fluorescence intensity (MFI) of CD73, CXCR3, PD-L1, LAP, HLADR, CD80, CD86 and CCR7. Antibodies were from BD (BD Company, NJ, USA) except FOXP3-PE (eBioscience, USA), CCL5-APC (R&D Systems, USA) and LAP-PE (Biosource, USA). We used FACS CantoII^TM^ analyzer, DIVA software (BD biosciences, USA), and the FlowJo software 9.1 (Tree Star, OR) for analysis. At least 300,000 events were acquired within the lymphocyte gate or within the AdMSC gate.

### Protein expression of MMP 2 and MMP9 upon AdMSC/PBMC interactions

We analysed the intracellular protein expression of MMP2 and MMP9 by FACS, in both AdMSC and T cells, alone, or following coculture, in the same conditions, using: anti-human MMP9-FITC antibody (Cat N. IC9111F, R&D Systems, Inc) and anti-human MMP2-PE antibody (Cat N. IC9023P, R&D Systems, Inc). We used FACS CantoII^TM^ analyzer, DIVA software (BD biosciences, USA), and the FlowJo software 9.1 (Tree Star, OR) for analysis. At least 300,000 events were acquired within the lymphocyte gate or within the AdMSC gate. We compared the expression in co-cultures to AdMSC or T cells alone.

### Effect of AdMSC/PBMC interactions on cytokine production

We evaluated IL-2, IL-4, IL-5, IL-10, IFN-γ and TNF-α production in coculture supernatants (days 1, 3 and 5), using Cytometric Bead Array (CBA Th1/Th2) (BD Company, USA), the FASCanto II (BD Company,USA), the BD FACS DIVA^TM^ program and the BD CBA Software (BD Company,USA). Concentration was calculated using the standard curves (FCAP Array Software BD Company,USA). Detection limits: 5 to 5000 pg/ml. For samples showing concentrations below the lower detection limit, we considered the value just below this limit to quantify cytokine production change in cocultures.

### Functional assays to evaluate molecules involved in AdMSC suppressive activity

We performed additional PBMC CFSE-labeled proliferation/suppression assays, combining inhibition of MMP, HLAG, IDO and CD73 activity to determine their contribution to AdMSC suppressive activity. We cocultivated AdMSC/PBMC with: IDO inhibitor-1-Metil-D-Triptophan (500 uM and 1000 uM; Sigma-Aldrich,St. Louis, USA), MMP2/9 inhibitor-SB-3CT (0.4 ug/ml and 4 ug/ml; Calbiochem Merck4Biosciences USA), CD73 inhibitor-Adenosin 5′(100 uM and 300 uM; Alpha, Beta-Metilen) Diphosphate (Sigma-Aldrich, St. Louis, USA), HLA-G inhibitor (0, 2 ug/ml and 2 ug/ml; Exbio Praha, Czech Republic), individually or in combination. We calculated T cell proliferation inhibition in the presence of AdMSC, with and without inhibitors, after 5 days of coculture, as previously.

### Statistical analysis

We used the following Statistical tests: Wilcoxon test for: AdMSC suppressive capacity over T cell proliferation, expression changes in the percentage and MFI (FACS), gene expression changes in T cells and AdMSC during PBMC/AdMSC interactions; One-Way ANOVA: to compare the % of proliferation inhibition in the 3 conditions tested; Spearman correlation test: for correlations among gene expression changes; Fisher’s exact test: for significance in the IPA network analyses; Mann-Whitney test: for differences in cytokine production during AdMSC/PBMC interactions, and AdMSC suppressive capacity with and without inhibitors of selected molecules. Significant differences: *(p < 0.05), **(p < 0.01) and ***(p < 0.001).

## Electronic supplementary material

Supplementary Info File
