# Supplementary material for: MMP9 integrates multiple immunoregulatory pathways that discriminate high suppressive activity of human mesenchymal stem cells
Source: Sci Rep. 2017 Apr 13;7:874. doi: 10.1038/s41598-017-00923-0 (PMC5429835; doi:10.1038/s41598-017-00923-0)
Supplement: Supplementary file 1 — Supplementary Info File [file 41598_2017_923_MOESM1_ESM.doc]

**MMP9 integrates multiple immunoregulatory pathways that discriminate high suppressive activity of human mesenchymal stem cells**

Carolina Lavini-Ramos^1, 2^, Hernandez Moura Silva^1^, ^3, 2^, Alessandra Soares-Schanoski^1, 2, 5^ , Sandra Maria Monteiro^1,2^, Ludmila Rodrigues Pinto Ferreira ^1, 2, 6^, Ana Paula Pacanaro^1, 2^, Samirah Gomes^4,7^, Janaína Baptista^1,2^, Kellen Faé^1, 2, 8^, Jorge Kalil^1, 2^, Verônica Coelho^1, 2, 4*^

^1^ Laboratory of Immunology, Heart Institute (InCor), School of Medicine, University of São Paulo, São Paulo, Brazil.

^2^ Institute for Investigation in Immunology, iii – INCT (National Institute of Science and Technology), São Paulo, Brazil.

^3^ Present affiliation: Molecular Pathogenesis Program, Kimmel Center for Biology and Medicine at the Skirball Institute, New York University School of Medicine, New York, NY 10016, USA

^4^ Center for Cellular and Molecular Therapy Studies (NETCEM), University of São Paulo, Brazil

^5^ Present affiliation: Biotechnology Center, Butantan Institute, São Paulo, Brazil

^6^ Universidade Santo Amaro (UNISA), São Paulo, Brazil

^7^Laboratory of Cellular, Genetic, and Molecular Nephrology, Renal Division, University of São Paulo, São Paulo, SP, Brazil

^8^Bacterial Vaccines Discovery & Early Development, Crucell Holland B.V., part of the Janssen pharmaceutical companies of Johnson and Johnson, Leiden, The Netherlands

^*^Address correspondence to: Verônica Coelho, MD, PhD

Laboratório de Imunologia

Instituto do Coração

Av Dr Enéas de Carvalho Aguiar, 44

bloco II, 9 andar - Cerqueira César

São Paulo - SP 05403-000

Tel: +55-11-2661 5905

Fax: +55-11-2661 5953

vecoelho@usp.br

**SUPPLEMENTARY FIGURE 1**

**
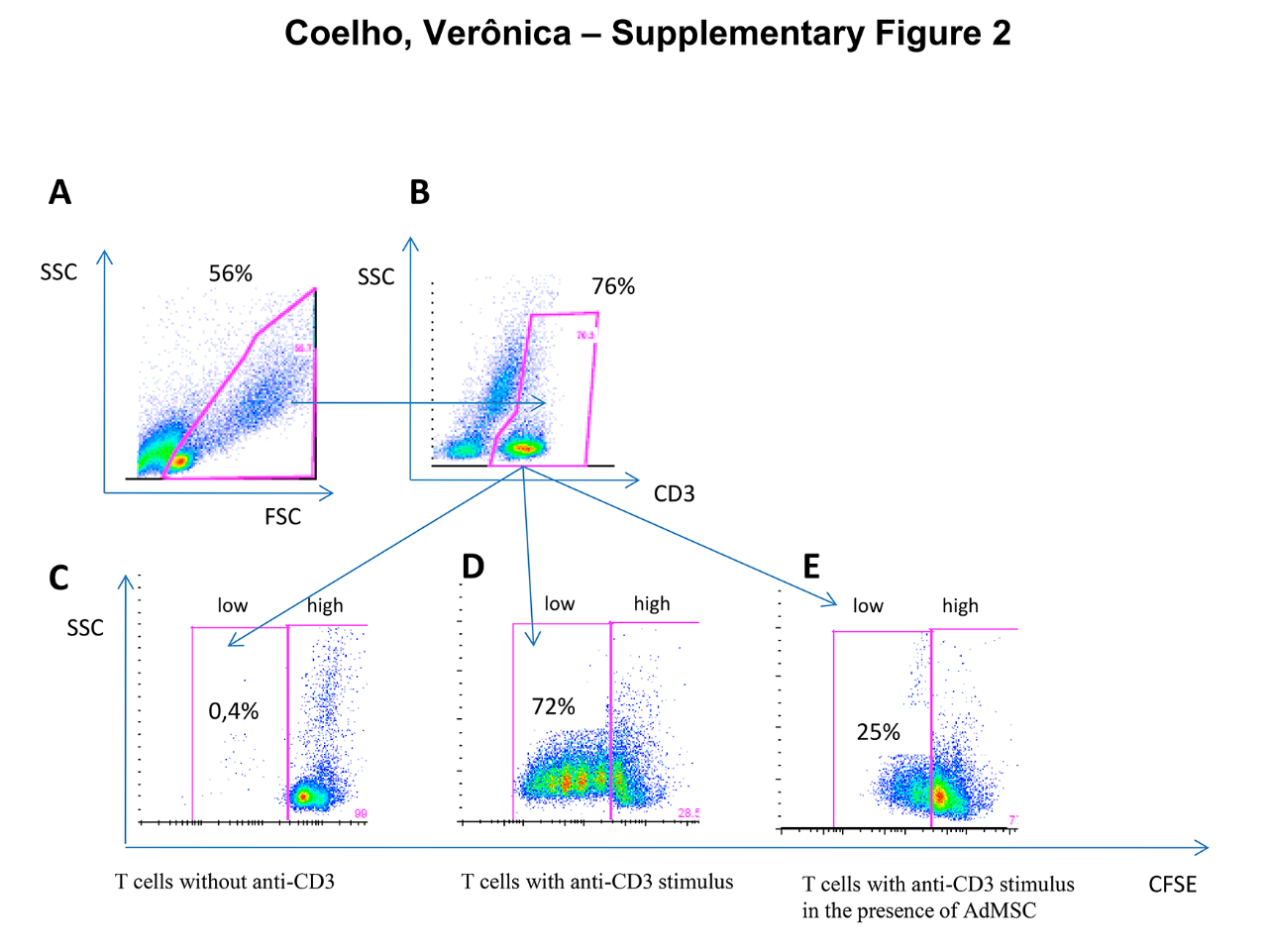
**

**SUPPLEMENTARY FIGURE 2**


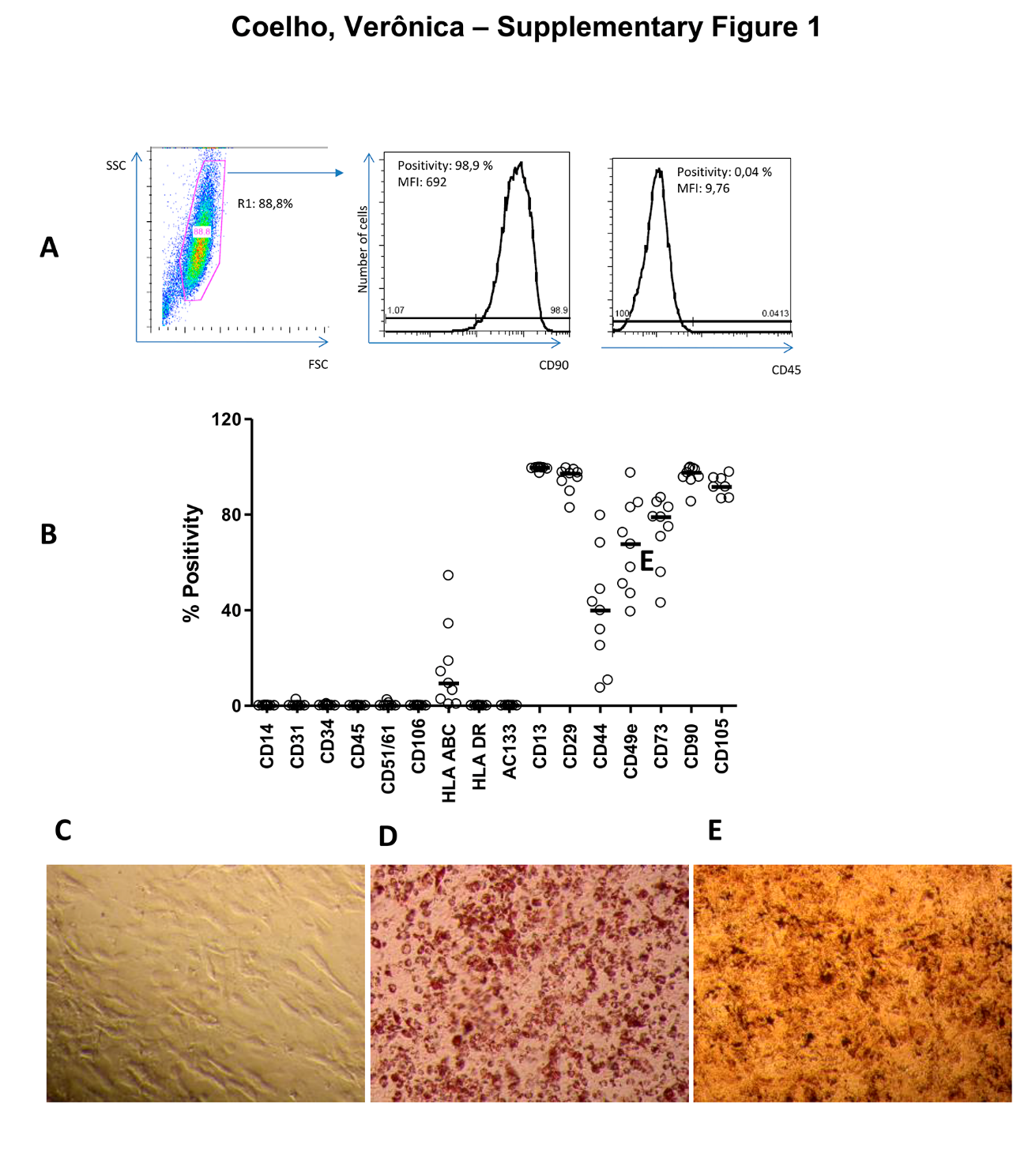


**SUPPLEMENTARY FIGURE 3**

**
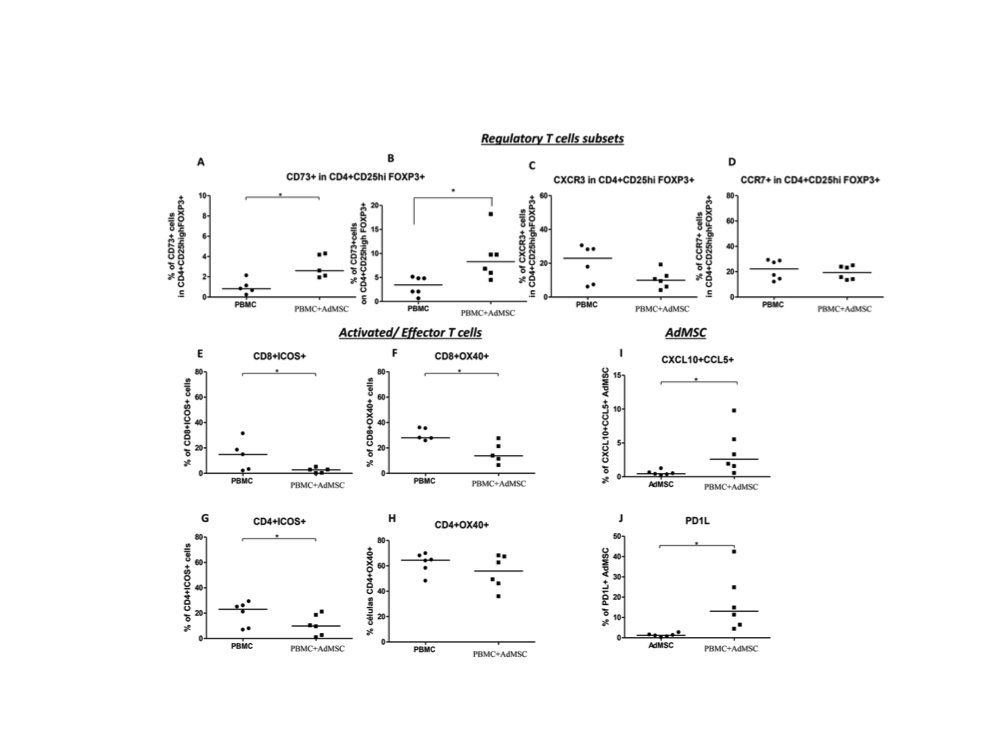
**

**SUPPLEMENTARY FIGURE 4**

**B**

**A**


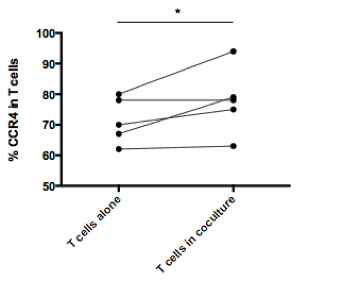

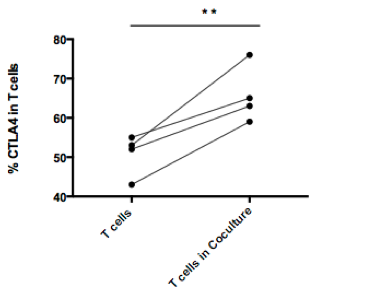


**FIGURES LEGEND**

**Supplementary Figure S1**. **Evaluation of AdMSC suppressive activity over T cell proliferation.** CFSE gate strategy. A representative analysis of a proliferation assay. Cells were first gated by FSCxSSC (A). Then, CD3^+^ cells we selected from PBMC (B) and the % of cells displaying low (cells that proliferated) or high (cells that did not proliferate) staining for CFSE was calculate in the condition without anti-CD3 stimulus (C), with anti-CD3 (D) and with anti-CD3 in the presence of AdMSC (E). The percentage of proliferation inhibition in the presence of AdMSC was calculated in comparison to the condition stimulated but with no AdMSC (D).

**Suppplemetary Figure S2**. **AdMSC immunophenotypic characterization**. (**A)** Strategy of analysis of AdMSC immunophenotypic characterization by Flow Cytometry. (**B)** AdMSC immunophenotypic characterization using a panel of molecules expressed or not on AdMSC (n=11 experiments). **C)** Untreated AdMSC. (**D)** AdMSC differentiation into adipocytes and **(E)** osteocytes. Increase 10x (n=4).

**Supplementary Figure S3. AdMSC/PBMC interactions induce/increase CD73+ Tregs and decrease activated effector T cells.** Cells were cocultured with anti-CD3 (1μg/ml) for 3 days and were collected for the evaluation of protein expression by flow cytometry (FACS CantoII flow cytometer, the program DIVA for acquisition and Flow Jo for analysis). A total of 300,000 events were analyzed within the lymphocyte gate selected by size (FSC) and granularity (SSC). We evaluated the effect of AdMSC/PBMC interaction on Treg subpopulations: (**A)** % of CD73+ on CD4 +CD25^hi^FOXP3 + cells after 5 days of coculture; (**B)** % of CD73+ on CD4 +CD25^hi^FOXP3 + cells after 8 days of coculture. We also evaluated the modulation of T cell activation by analyzing the % of ICOS and OX40 within the population of CD4 + and CD8 + cells; (**C)** % of CD8+ ICOS+ cells; (**D)** % of CD4+ ICOS+ cells; (**E)** % of CD8+ OX40+ cells; all after 5 days of coculture. On AdMSC (CD90+) we evaluated: (**F)** % of CXCL10+CCL5+ cells; (**G)** % of PD1L. n = 6, except for CD8+ analysis, n= 5. Paired t-test Wilcoxon. *p<0.05

**Supplementary Figure S4. Protein expression of CCR4 and CTLA4 in T cells.** Increase in the percentage of T cells expressing CCR4 (A) and CTLA4 (B), before and following coculture of AdMSC and PBMC Paired t test * p= 0.04 and ** p=0.007 respectively.

**Supplementary Table 1. Genes evaluated for mRNA expression in AdMSC after PBMC/AdMSC interactions in suppressive assays.** Gene expression evaluation by Real Time PCR in triplicate in purified T cells from PBMC stimulated with anti-CD3 after 3 days of culture with or without AdMSC. Genes were classified as predominantly immunoregulatory (REG) (in bold) or proinflammatory (INFLAMMA) (underlined).


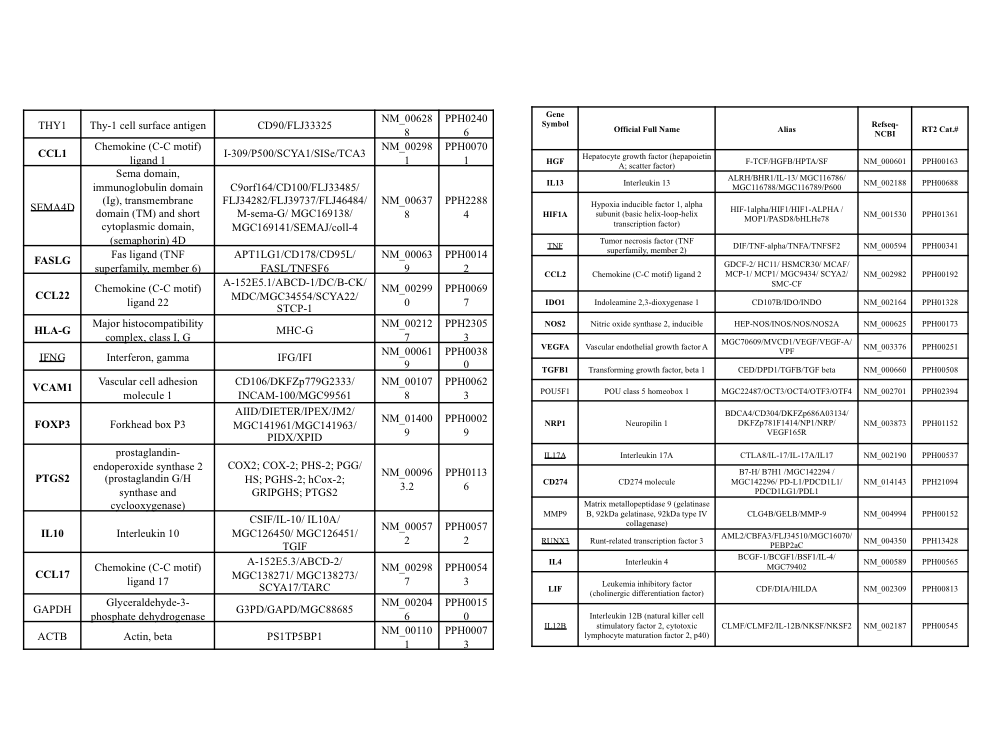


**Supplementary Table 2.** **Genes evaluated for mRNA expression in T cells after PBMC/AdMSC interactions in suppressive assays.** Gene expression evaluation by Real Time PCR in triplicate in purified AdMSC cells stimulated with anti-CD3 after 3 days of culture with or without PBMC. Genes were classified as predominantly immunoregulatory (REG) (in bold) or proinflammatory (INFLAMMA) (underlined).


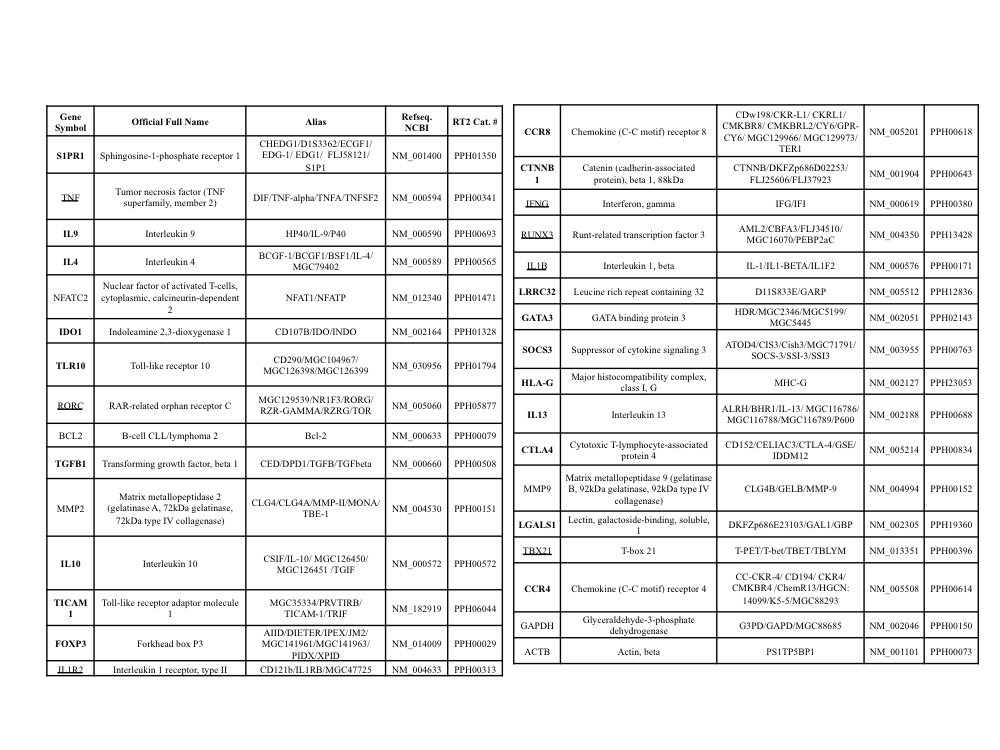


Supplementary Table 3. List of Networks with higher scores built for AdMSC (purple) and T cells (blue) gene expression data. The list of differentially expressed genes (⭡upregulated or ⭣downregulated) in AdMSC and T cells were mapped to their corresponding gene objects in Ingenuity Knowledge Base (IKB). These “focus molecules” were algorithmically selected and scored, a score ≥ 2 indicated a probability p ≤ 0.01 that the focus genes in a network were found together by chance. The software built 4 networks for the list of genes expressed in LT (blue) and 7 networks for the list of genes altered in AdMSC (purple). Highlighted in yellow the two networks (Number 5 for MSC e 2 for T cells) with greater number of nodes in common (18 nodes) as represented in figure 6.

| **ID** | **Analysis** | **Molecules in network** | **Score** | **Focus molecules** | **Top diseases and Functions** |
| --- | --- | --- | --- | --- | --- |
| **1** | **T cells** | Akt, **⭡BCL2**, BCR (complex), Calcineurin protein(s), **⭡CTLA4, Fcer1, ⭡FOXP3, Gm-csf, Ifn, Ifn gamma, Ifnar, Ige,** IgG, IgG1, IgG2a, Igm, Ikb, ⭡IL9, IL23, IL12 (complex), IL12 (family), Il8r, Immunoglobulin, Interferon alpha, JAK,MAP2K1/2, NFAT (complex), Nfat (family), **⭡SOCS3,** STAT5a/b, TCR, TH2 Cytokine, Tlr, **⭡TLR10,** Tnf (family) | 15 | 6 | Inflammatory Disease, Respiratory Disease, Cell-To-Cell Signaling and Interaction |
| **1** | AdMSC | Aconitase, Adaptor protein 1, C/ebp, **⭡CCL1**, **⭡CCL17**, **⭡CD274**, CD80/CD86, Cebp, cyclooxygenase, Fc gamma receptor, Fcer1, Ferritin, Gm-csf, Hat, HLA-DQ,HLA-DR, **⭡IDO1**, IFN alpha/beta, Ifn gamma, Ifnar, Ikb, IL23, IL-1R, IL12 (complex), Il12 receptor, IL17a dimer, lymphotoxin-alpha1-beta2, MHC Class I (complex), NFkB (family), NfkB-RelA, NfkB1-RelA, SAA, Tenascin, **⭡TNF**, Tnf receptor | 10 | 5 | Antimicrobial Response, Cellular Movement, Hematological System Development and Function |
| **2** | MSC | AChR, ALT, **⭡CCL2**, **⭡CCL22**, **⭡FOXP3**, HISTONE, **⭡HLA-G**, IFN Beta, IFN type 1, Iga, Ige, IgG, IgG1, Igg2, Igg3, IgG2a, IgG2b, Igm, **⭡IL10**, IL12 (family), Immunoglobulin, Interferon alpha, JAK,MHC,MHC CLASS I (family), NFAT (complex), NFkB (complex), p70 S6k, SCAVENGER receptor CLASS A, STAT, SYK/ZAP, TCR, TH1 Cytokine, Tlr, VAV | 10 | 5 | Cellular Movement, Hematological System Development and Function, Immune Cell Trafficking |
| **2** | T cells | Alp, Alpha catenin, apyrase, Collagen type I, Collagen type II, Collagen(s), elastase, ERK1/2, Fc gamma receptor, Fgf, Fibrin, Fibrinogen, gelatinase, Growth hormone, HDL, HLA-DR, Hsp27, IDO1, IFN Beta, IFN type 1, IL1, Integrin, Laminin, LDL, **⭡MMP2**, **⭡MMP9**, NFkB (family), NfkB-RelA, Pdgf (complex), PDGF BB, PI3K (family), Pld, PP2A, SAA, Tgf beta | 6 | 3 | Carbohydrate Metabolism, Small Molecule Biochemistry, Inflammatory Response |
| **3** | MSC | 26s Proteasome, Acot1, CAK, chemokine, Ck2, DCT, Fcr, GBP7, GPR19, Gsk3, HRH3, **⭡IDO1**, Ifi47, **⭡IFNG**, immune complex, Jnk, Metalloprotease, mir-150, mir-375, Mmp, MTORC1, Pdgfr, PI3Kγ, Pka, **⭡POU5F1**, **⭡PTGES2**, RHBDD3, RNA polymerase II, Sfk, SLC52A2, Slfn1, Smad2/3, **⭣TGFB1**, TNFSF18, Vegf | 10 | 5 | Amino Acid Metabolism, Small Molecule Biochemistry, Cellular Movement |
| **3** | T cells | AMPK, Ap1, CD3, Cg, Creb, **⭡CXCL8**, ERK, estrogen receptor, FSH, Gsk3, Hdac, HISTONE, Histone h3, Histone h4, Hsp70, IKK (complex), IL1B, Insulin, Lh, **⭡LRRC32**, Mapk, Mek, NFkB (complex), Nr1h, P110, p85 (pik3r), PI3K (complex), Pkc(s), Pro-inflammatory Cytokine, Ras, Ras homolog, RNA polymerase II, TCF, TSH, Vegf | 6 | 3 | Cardiovascular System Development and Function, Cellular Movement, Hematological System Development and Function |
| **4** | MSC | Akt, AMPK, arginase, calpain, caspase, Cyclin A, Cyclin E, cytochrome C, DNA-methyltransferase, **⭡FASLG**, GOT, Hdac, HDL, histone deacetylase, Histone h4, Ifn, Il8r, MHC Class II (complex), MHC II, Mlc, MTORC2, N-cor,NADPH oxidase, Nos, **⭡NOS2**, Notch, Nr1h, PARP, PEPCK, PRKAA, Pro-inflammatory Cytokine, Rb, **⭡RUNX3**, TH2 Cytokine, Tnf (family) | 6 | 3 | Cellular Movement, Cellular Development, Hematopoiesis |
| **4** | T cells | 26s Proteasome, AMBP, ASS1, B3GNT2, C/ebp, CD6, Cpla2, CR1L, CRHR1, Cyclin E, cyclooxygenase, ERK1/2, Focal adhesion kinase, G protein alphai, GDF15, IL17B, IL36A, IRAK, Jnk, KLRB1, LUM, mir-23, Mmp, **⭡MMP9**, myosin-light-chain kinase, Neurotrophin, P38 MAPK, S100A12, SEMA3A, Sfk, sphingomyelinase, SRC (family), TNFAIP8L2, Tni, UCN | 2 | 1 | Hematological System Development and Function, Inflammatory Response, Tissue Morphology |
| **5** | MSC | Alp, C1q, collagen, Collagen Alpha1, Collagen type I, Collagen type II, Collagen type IV, Collagen(s), Creb, ERK1/2, Fcgr3, Fibrinogen, gelatinase, Growth hormone, HLA Class I, Hsp27, IL1, Integrin, Laminin, Laminin1, LDL, Mek, **⭡MMP9**, N-Cadherin, Pdgf (complex), PDGF BB, PI3K (family), Rap1, Secretase gamma, Smad, Sphk, STAT5a/b, Tgf beta, **⭣THY1**, Timp | 3 | 2 | Cancer, Gastrointestinal Disease, Respiratory Disease |
| **6** | MSC | Actin, Alpha catenin, Ap1, CaMKII, CD3, Cg, cytochrome-c oxidase, ERK, Erm, estrogen receptor, Focal adhesion kinase, G protein alphai, Histone h3, Hsp70, Hsp90, IKK (complex), Insulin, **⭡LIF**, MAP2K1/2, Mapk, Nfat (family), P38 MAPK, p85 (pik3r), PI3K (complex), Pkc(s), PLA2, Proinsulin, Rac, Ras, Ras homolog, Rock, Sapk, Sod, SRC (family), **⭡VCAM1** | 3 | 2 | Cellular Movement, Embryonic Development, Cell-To-Cell Signaling and Interaction |
| **7** | MSC | CD72, CD80, CD40LG, HLA-DQB1, IGHG1, IL4, IL12 (complex), Immunoglobulin, MET,miR-1225-5p (miRNAs w/seed UGGGUAC), miR-3090-3p (and other miRNAs w/seed CCCAGGU), miR-3169 (miRNAs w/seed AGGACUG), miR-3939 (miRNAs w/seed ACGCGCA), miR-4258 (miRNAs w/seed CCCGCCA), miR-4449 (miRNAs w/seed GUCCCGG), miR-4460 (miRNAs w/seed UAGUGGU), miR-4724-5p (miRNAs w/seed ACUGAAC), miR-4733-3p (and other miRNAs w/seed CACCAGG), miR-487b-3p (miRNAs w/seed AUCGUAC), miR-513a-5p (miRNAs w/seed UCACAGG), miR-647 (miRNAs w/seed UGGCUGC), Mlc, MST1R, PLCG1, PLCG2, PLXNB1, PLXNB2, PTPN1, PTPRC, Rac, RAPH1, RRAS, SEMA4, **⭡SEMA4D**, Timd2 | 2 | 1 | Cell-To-Cell Signaling and Interaction, Hematological System Development and Function, Humoral Immune Response |
